# The fate of endemic insects of the Andean region under the effect of global warming

**DOI:** 10.1371/journal.pone.0186655

**Published:** 2017-10-16

**Authors:** Sara I. Montemayor, María Cecilia Melo, María Celeste Scattolini, Martina E. Pocco, María Guadalupe del Río, Gimena Dellapé, Erica E. Scheibler, Sergio A. Roig, Carla G. Cazorla, Pablo M. Dellapé

**Affiliations:** 1 Universidad Nacional de La Plata, CONICET, División Entomología, Museo de La Plata, Paseo del Bosque s/n B1900FWA, La Plata, Buenos Aires, Argentina; 2 Centro de Estudios Parasitológicos y de Vectores (CEPAVE), CCT La Plata CONICET–Universidad Nacional de La Plata (UNLP), Boulevard 120 e/60 y 64 S/N, La Plata, Argentina; 3 Laboratorio de Entomología, IADIZA (CCT CONICET-Mendoza), Avda, Ruiz Leal s/n, Parque Gral, San Martín, Mendoza, Argentina; 4 Universidad Nacional de La Plata, CONICET, División Entomología, Museo de La Plata, ANEXO MUSEO, calle 122 y 60, La Plata, Buenos Aires, Argentina; Universita degli Studi di Napoli Federico II, ITALY

## Abstract

Three independent but complementary lines of research have provided evidence for the recognition of refugia: paleontology, phylogeography and species distributional modelling (SDM). SDM assesses the ecological requirements of a species based on its known occurrences and enables its distribution to be projected on past climatological reconstructions. One advantage over the other two approaches is that it provides an explicit link to environment and geography, thereby enabling the analysis of a large number of taxa in the search for more general refugia patterns. We propose a methodology for using SDM to recognize biogeographical patterns of endemic insects from Southern South America. We built species distributional models for 59 insect species using Maxent. The species analyzed in the study have narrow niche breadth and were classified into four assemblages according to the ecoregion they inhabit. Models were built for the Late Pleistocene, Mid-Holocene and Present. Through the procedure developed for this study we used the models to recognize: Late Pleistocene refugia; areas with high species richness during all three periods; climatically constant areas (*in situ* refugia); consistent patterns among *in situ* refugia, Pleistocene refugia and current distribution of endemic species. We recognized two adjacent Pleistocene refugia with distinct climates; four *in situ* refugia, some of which are undergoing a process of fragmentation and retraction or enlargement. Interestingly, we found a congruent pattern among *in situ* refugia, Pleistocene refugia and endemic species. Our results seem to be consistent with the idea that long-term climate stability is known to have a key role in promoting persistence of biodiversity in an area. Our Pleistocene and *in situ* refugia are consistent with refugia identified in studies focusing on different taxa and applying other methodologies, showing that the method developed can be used to identify such areas and prove their importance for conservation.

## Introduction

Recognizing refugia is crucial for understanding the evolutionary history of world’s biodiversity and protecting it against climate change because refugia play a major role in determining current and future diversity patterns [1]. Refugia are places which, under unfavorable climatic conditions, remain milder and more or less constant and where components of biodiversity can retreat to, persist in or potentially expand from to the surrounding landscape [2] over long evolutionary time-scales [1]. Once climatic conditions improve, many species with narrow climatic tolerances remain restricted to these areas and are unable to migrate out of them [3]. Refugia thus have higher species richness and a relatively higher number of endemic species and relict lineages than the neighboring landscape ([4] and references therein).

Three independent but complementary lines of research have provided evidence for the recognition of refugia: paleontology, phylogeography, and species distributional modelling (SDM) [5]. Paleontology was the first approach used to recognize refugia, but only provides a partial picture of the past because of the incomplete geological record [6, 5]. The phylogeographical approach is the most widely used for identifying refugia for single species [1]. SDM has been used in several recently published studies [7]. This methodology assesses the ecological requirements of a species based on its known occurrences and allows its distribution to be projected on past climatological reconstructions [1, 5, 7]. One advantage over the other two approaches is that it provides an explicit link to environment and geography, thereby enabling the analysis of a large number of taxa in the search for more general refugia patterns.

Insects encompass most of the terrestrial fauna both in terms of biomass and diversity. Moreover, they are widely distributed and present in almost all ecosystems [8]. Nevertheless, little is currently known regarding how climate change is impacting their distribution. Few studies have analyzed evolutionary patterns related to climate change across multiple species at regional scale. Fossil evidence of Pleistocene insects exhibits great morphological similarity to their modern equivalents, moreover, species assemblages have remained similar over time [9]. An example of this is the fossil Pleistocene Coleoptera (18–15 ka) assemblage in the Lakes Region in Chile, which includes the same species as it does today ([10] and references therein). Morphological stasis can be explained by the fact that insect species track their climatic niche so that the environmental conditions in which they live remain similar over long periods of time [9]. Niche conservatism over time is a key assumption for predicting the potential distribution of species in the past. Hence, insects are an excellent group to use for inferring refugia using the SDM approach.

The study area is the southernmost part of South America. This region has been shaped over the past million years by geological and climatic events such as orogeny, volcanism, cyclic ice ages and sea level fluctuations along the Atlantic coast [11, 12]. Pleistocene glaciations (1.8 Ma–10 ka) significantly altered the landscape in this region, mainly by extension of the ice shield and shifts in climate and sea level [13]. The many glacial advances and retreats during the Pleistocene, such as the Last Glacial Maximum (LGM 20–18 ka), may have directly affected populations located in the Andean region [14].

In this study, we propose an iterative methodology using SDM for recognizing: i) climatically suitable areas for hosting a large number of species during the Late Pleistocene, Mid-Holocene and Present; ii) climatically stable areas over time; and iii) refugia. We applied this new approach to endemic species assemblages of the Andean Region (59 species) considering that there are few studies on either the insect taxa analyzed or the study area.

## Materials and methods

### Study area

The study area is located in the southernmost part of South America, between 23°/55° S and 65°/76° W, corresponding to the Andean Region [15]. The climate ranges from cold temperate to subpolar, with harsh conditions and large areas where the climate is either very cold or extremely dry [16]. During the LGM the physiognomy of the eastern Andean region was dominated by open vegetated areas, mostly grasslands, steppe and cold steppe biomes, while the western Andean region had areas (between 40° and 42°) characterized by Magellanic forest and moorlands [17–18].

We centered our analyses on the terrestrial ecoregions, following the WWF hierarchical classification of ecoregions [19]. The study area includes four ecoregions: the Valdivian Temperate Forest (VTF), the Chilean Matorral (CM), the Patagonian Steppe (PS) and the Magellanic Subpolar Forest (MSF).

The VTF covers a narrow continental strip between the western slope of the Andes and the Pacific Ocean, running from 35° to 48° S. It represents a unique assemblage of ancient Gondwanaland relict species and hosts many endemic species.

The CM constitutes a 100 km-wide strip extending along the central part of the Chilean coast. This ecoregion represents the transitional habitat between the ultra-dry Atacama Desert to the north, and the moist Valdivian temperate forests to the south; it has various endemic plant species with affinities to the tropics, the Antarctic and the Andes.

The PS extends roughly from the mid-Andean Precordillera southward, ending just north of the Straights of Magellan near the River Gallegos. It extends north-west as shrubland steppe and to the north gradually making the transition to the Argentinean Monte. It has high levels of endemism in both plants and animals.

The MSF extends along the Pacific coast and foothills of southernmost Chile and southwestern Argentina. It was covered by glaciers during the last ice age, and the landscape is deeply dissected by fjords, with numerous islands, inlets and channels. It is dominated by trees of the genus *Nothofagus* and its fauna is related to that of the bordering ecoregions, especially the Valdivian temperate forests and the Patagonian steppe. Nevertheless, it is inhabited by unique and endemic animal and plant species.

### Species data

We used 68 insect species endemic to the Andean Region, belonging to the orders Coleoptera (4 spp.), Diptera (18 spp.), Hemiptera (20 spp.), Odonata (7 spp.) and Orthoptera (19 spp.). For each species, a presence-only database was compiled from the literature and specimens deposited in the entomological collections of Museo de La Plata (Buenos Aires, Argentina) and Instituto Argentino de Investigaciones de Zonas Áridas- IADIZA (Mendoza, Argentina). Datasets were generated by experts on the groups in order to secure trustworthy information. We eliminated any duplicated records, generating a dataset of 1,070 records (S1 Table). Then we conducted Moran’s I test at multiple distance classes using SAM 4.0 [20] to test whether the dataset had spatial autocorrelation biases, and if so, we removed localities one by one until the autocorrelation was eliminated, endeavoring to maintain the maximum number of localities possible. This procedure has been recently frequently used (e.g, [21–22]).

### Recognition of species assemblages

Terrestrial ecoregions have been identified on the basis of climate, flora, fauna, and physiography [19] and represent a solid approach to recognizing species assemblages nested by similar environmental requirements. Thus, each species was classified into an assemblage considering the ecoregion where most of its known records are distributed, or by its current potential distribution. We recognized four assemblages: 35 species for the Valdivian Temperate Forest (VTF), ten for the Chilean Matorral (CM), 12 for the Patagonian Steppe (PS), and 11 for the Magellanic Subpolar Forest (MSF).

### Environmental variables and species distributional modeling

We modeled present and past climatic conditions from the Late Pleistocene (LGM 22 ky) and Mid-Holocene (6 ky) from the fully known present distribution of the species. To build the models, we used the set of 19 bioclimatic variables available at WorldClim database [23] with 2.5 minute (~5 km^2^) spatial resolution. For the past we used the CCSM4 General Circulation Model (GCM) with the same spatial resolution as for the present. The models were performed using Maxent 3.3.3k [24]. There are several methods to detect collinearity, but they are arbitrary and there is no detailed ecological studies to determine which variables should be excluded from the analysis. Maxent [24] implements a form of regularization that can exclude variables from the final model, thereby eliminating the requirement for prior variable selection [25]. It implicitly deals with feature selection and is unlikely to be improved and more likely to be degraded by procedures that use other modelling methods to pre-selected variables (e.g., [26]). Moreover, in macroecological studies dealing with several species it has been recommended to build the models with the full dataset [27], tuning the settings to avoid possible overfitting, using fewer feature classes and stronger regularization [26–28]. Considering all these and following [28], we tuned/set the regularization multiplier to 2 and used the hinge feature class, since it has proved to give the best results for species with less than 25 records [28–31]. Maxent outputs were converted into binary maps based on the ‘minimum training presence logistic threshold’, which indicates values above which the climate conditions are suitable for the survival of the modelled species. We used this threshold because it guarantees that all possible presences of the target species are predicted as suitable [29].

### Model validation

We used the jackknife validation methodology [29] to evaluate the predictive accuracy of our model, which was specifically designed for a small number of occurrences. This approach is based on removing one locality point from the dataset and building a model using the remaining n–1 localities. The ability of each n-1 model to predict the locality excluded is tested. To do this, the n-1 models need to be converted into binary presence–absence maps using a threshold. We used the ‘minimum training presence logistic threshold’, which is the most restrictive because it is the lowest value of the prediction for any of the presence records. As many n-1 models are built as point localities exist. The significance of the models is tested using the P value program [29], if P ≤ 0.05 the model is validated.

### Niche breadth

To identify species that are good characterizers of the climatic space of their ecoregion, species with narrow climatic niche breadth were selected. We calculated Levin’s concentration metrics (implemented in ENMTOOLS 1.3) [32] of the current average models (which are functions of the 19 climatic variables). This index ranges from 0 (indicating narrow niche breadth) to 1 (indicating wide niche breadth) [33], and we consider values ≤ 0.5 as specialist taxa.

### Identification of species in expansion or retraction and construction of Late Pleistocene refugia maps (PRMs)

To recognize whether species distributions were constant, retracting or expanding over time, we counted the number of pixels coded as presences from both Late Pleistocene and Present binary maps. We used this information to calculate the distributional percentage of retraction/expansion per species. To build these maps (PRMs), we considered the species of each assemblage whose distributions were narrower in the Late Pleistocene. The binary Pleistocene maps were added, retaining the areas where most of the species overlap (80% or more of each assemblage). We excluded the LGM Patagonian ice sheet from these maps [34] because we were searching for areas in which species occur over time. The raw data from the rasters of the current 19 variables were extracted for the area occupied by the refugia. To analyze climatic differences between refugia, Kruskal- Wallis’s tests were performed for the 19 bioclimatic variables and boxplots were built using this information to compare climatic differences between refugia.

### Insect-rich area maps (IAMs) and in situ refugia maps (IRMs)

To identify areas where a wide array of the species of each assemblage could have lived during the different time periods, we built “Insect-rich area maps” (IAMs). We then identified the region where these areas have remained climatically constant throughout all three periods (Late Pleistocene, Mid-Holocene and Present) in “*in situ* refugia maps” (IRMs). For the IAMs we added the binary maps of the species of each assemblage according to time period. To build the IRMs for each assemblage, we superimposed the IAMs for the three periods to recover the suitable areas that have remained in the same geographical space over time. In the IAMs and IRMs we retained the areas where most of the species (80% or more) for each ecoregion find suitable conditions for survival. All these maps were developed using QGIS 2.8.2.

### Consistency analysis of in situ refugia, Pleistocene refugia and distributions of endemic species

Distributional patterns of endemic species have been used to infer the locations of other environmentally meaningful areas such as refugia ([3], and references therein). To analyze whether there is a consistent pattern among the *in situ* refugia, Pleistocene refugia and current distribution of endemic species, we selected the Valdivian Temperate Forest assemblage, as it has several endemic species (nine). We built an endemic species map (ESM) by adding the present binary maps of the nine species, retaining the areas where more than 80% of these species coincide. Then we built an IRM as explained previously but excluding the nine endemic species in order to avoid redundant evidence. Finally, we superimposed the IAM with the IRM and the PRM in search of patterns.

## Results

### Species distributional modeling and model validation

Of the 68 species initially considered, nine were discarded from the subsequent analyses for the following reasons: models were not validated by the jackknife approach [*Sigara* (*T*.) *vuriloche* (Bachmann) and *Phorbanta variabilis* (Signoret) (Hemiptera)]; niche breadth analysis did not identify them as specialist species [*Rhaptus cuadricollis* (Spinola) (Hemiptera)]; and suitable climatic areas were not identified for their existence in the past [*Stilobezzia* (*A*.) *rava* Ingram & Macfie (Diptera); *Sigara vereertbruggheni* Hungerford; *Ea australis* Distant (Hemiptera) and *Bufonacris terrestris* Walker, *Nahuelia rubriventris* Liebermann, *Tebacris nigrisoma* Cigliano (Orthoptera)].

As a result, we retained 59 species for the final analyses. They were distributed among four species assemblages: Valdivian Temperate Forest (VTF) (31 spp.), Chilean Matorral (CM) (10 spp.), Patagonian Steppe (PS) (8 spp.) and Magellanic Subpolar Forest (MSF) (10 spp.).

### Expansion/retraction of the potential distributions of the species

Of the 59 species analyzed, 33 showed retraction of their potential distribution from the Late Pleistocene to the Present (Table 1), 15 species expanded their distribution, and 11 species remained in a similarly sized area over time. Species of all the orders are in a process of distributional retraction: Coleoptera (3 spp.), Diptera (6 spp.), Hemiptera (10 spp.), Odonata (4 spp.) and Orthoptera (10 spp.). Since the Late Pleistocene these species have lost potentially suitable territory by about 21–67% (Coleoptera), 10–41% (Diptera), 10–88% (Hemiptera), 14–48% (Odonata) and 15–83% (Orthoptera). Most of the species in three of the assemblages are retracting [VTF (55%), CM (80%) and PS (75%)], whereas most of the species in MSF are undergoing a process of expansion (80%). The area of potential distribution for 7 of the 13 species endemic to an ecoregion has retracted since the Late Pleistocene.

**Table 1:** Areal distribution changes of the study species between Late Pleistocene and Present.

Order	Species retracting to the Present	Species expanding to the Present	Without major modifications
**Coleoptera**	*D*. *hyrtella* (52%)-MSF	* *	*H*. *tuberculifer-* endemic to VTF
** **	*H*. *roseus* (67%) endemic to VTF	* *	* *
** **	*M*. *spinifer* (21%)-endemic to VTF	* *	* *
**Diptera**	*D*. *andensis* (40%)- PS	*A*. *obnubilis* (20%)-VTF	*P*. *mapuche*-VTF
** **	*F*. *(F*.*) fusca* (10%)-VTF	*A*. *obfuscatus* (60%)-VTF	*S*. *(A*.*) curvistyla* (3%)-endemic to VTF
** **	*F*. *(F*.*) multipicta* (41%)-VTF	*D*. *monticola* (74%)-VTF	*S*. *(A*.*) furva* (3%)-endemic to VTF
** **	*F*.*(E*.*) setosicrus* (29%)-VTF	*F*. *(F*.*) chilensis* (38%)-MSF	*S*. *(A*.*) borkenti* (3.5%)-VTF
** **	*S*. *patagonica* (24%)-VTF	*S*. *(A*.*) bicinctipes* (70%)-endemic to VTF	*D*. *shannoni* (3%)-VTF
** **	*P*. *subfuscula* (34%)-endemic to VTF	*S*. *(A*.*) varia* (58%)-VTF	* *
**Heteroptera**	*A*. *cumingii* (51%)-VTF	*D*. *punctiventris* (11%)-MSF	* *
** **	*B*. *atrata* (75%)-VTF	*I*. *insularis* (17%)-MSF	* *
** **	*B*. *polychroma* (10%)-MSF	*S*. *poecilus* (13%)-MSF	* *
** **	*C*. *plana* (29%)-VTF	*S*. *perpunctata* (77%)-MSF	* *
** **	*P*. *gayi* (48%)-VTF	*P*. *bergi* (25%)-MSF	* *
** **	*S*. *humeralis* (31%)-CM	* *	* *
** **	*E*. *(E*.*) quadrata* (53%)-PS		* *
** **	*S*. *(T*.*) egbertae* (88%)-VTF	* *	* *
** **	*S*. *(T*.*) jensenhaarupi* (29%)-PS	* *	* *
** **	*N*. *(P*.*) virescens* (38%)-CM	* *	* *
**Odonata**	*A*. *peterseni* (48%)-PS	*R*. *variegata* (10%)-MSF	*P*. *raptor*-VTF
** **	*C*. *interruptum* (14%)-PS	* *	*S*. *villosum* endemic to VTF
** **	*G*. *paradoxa* (30%)-VTF	* *	
** **	*N*. *punctata* (48%)-endemic to VTF	* *	* *
**Orthoptera**	*A*. *bullocki* (43%)-VTF	*N*. *fictor* (19%)-PS	*E*. *lutescens*-CM
** **	*A*. *eumera* (29%)-CM	*T*. *willemsei* (61%)-endemic to PS	*M*. *cinerascens*-CM
** **	*B*. *bruchi* (52%)-PS	*T*. *magellanica* (61%)-MSF	* *
** **	*C*. *sulcaticollis* (23%)-CM	*T*. *angusticollis* (23%)-VTF	* *
** **	*E*. *wagenknechti* (15%)-endemic to CM	* *	* *
** **	*E*. *ensicornis* (34%)-VTF	* *	* *
** **	*M*. *nigripes* (43%)-VTF	* *	* *
** **	*T*. *chilensis* (39%)-endemic to CM	* *	* *
** **	*T*. *sergioi* (33%)-CM	* *	* *
** **	*P*. *recutita* (83%)-endemic to CM	* *	* *

List of the species that are retracting/expanding their potential distribution from the Late Pleistocene to the Present.

### Late Pleistocene refugia

**Fig 1:**
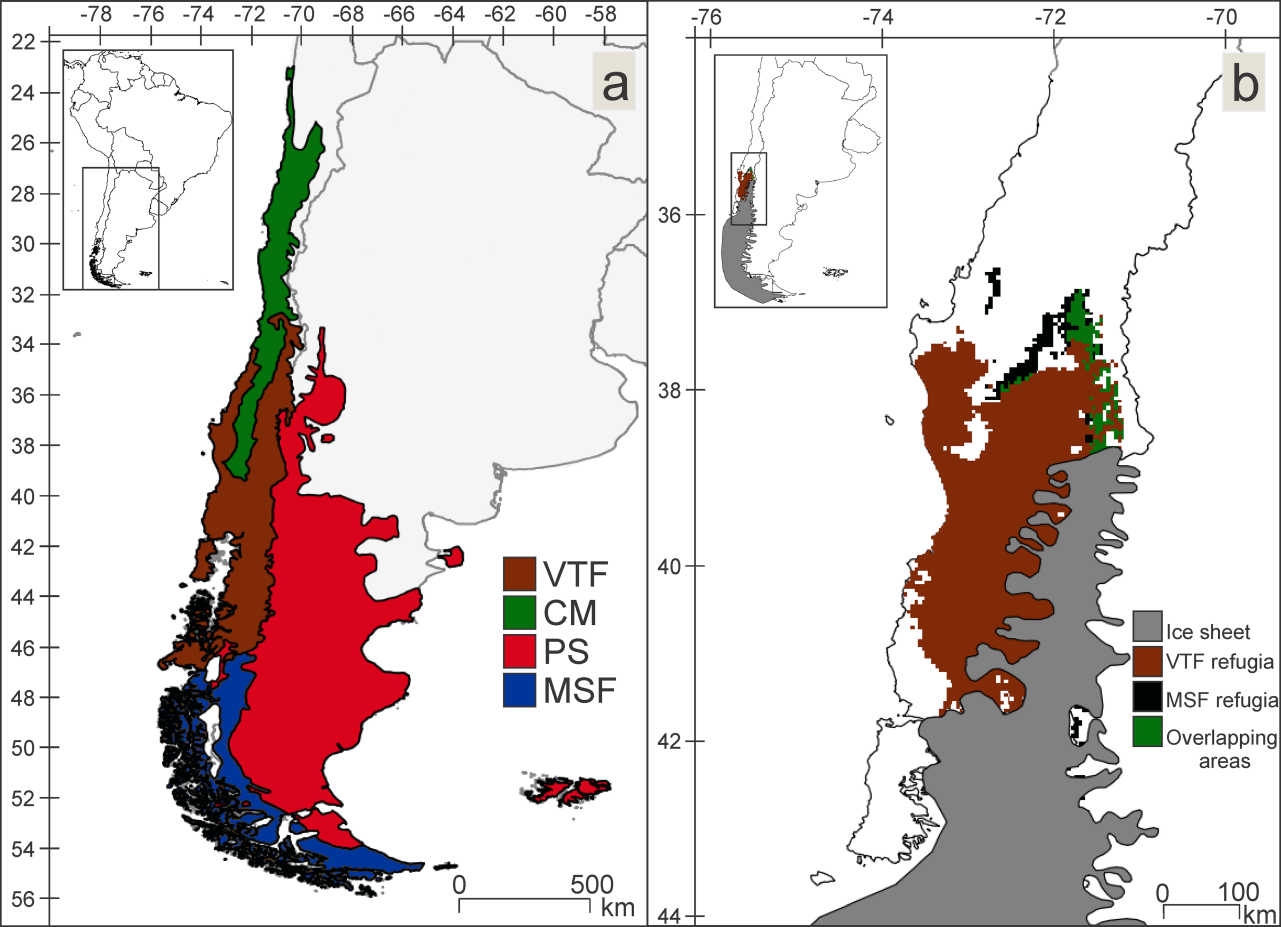
Ecoregions and resulting Pleistocenic refugia. (a) Ecoregions: Brown- Valdivian Temperate Forest (VTF), green- Chilean Matorral (CM), red- Patagonian Steppe (PS), blue- Magellanic Subpolar Forest (MSF). (b) Late Pleistocenic refugia: Brown- Valdivian Temperate Forest (VTF) refuge, black- Magellanic Subpolar Forest (MSF) refuge, green- overlapping areas between the two refugia, grey- ice sheet.

**Fig 2:**
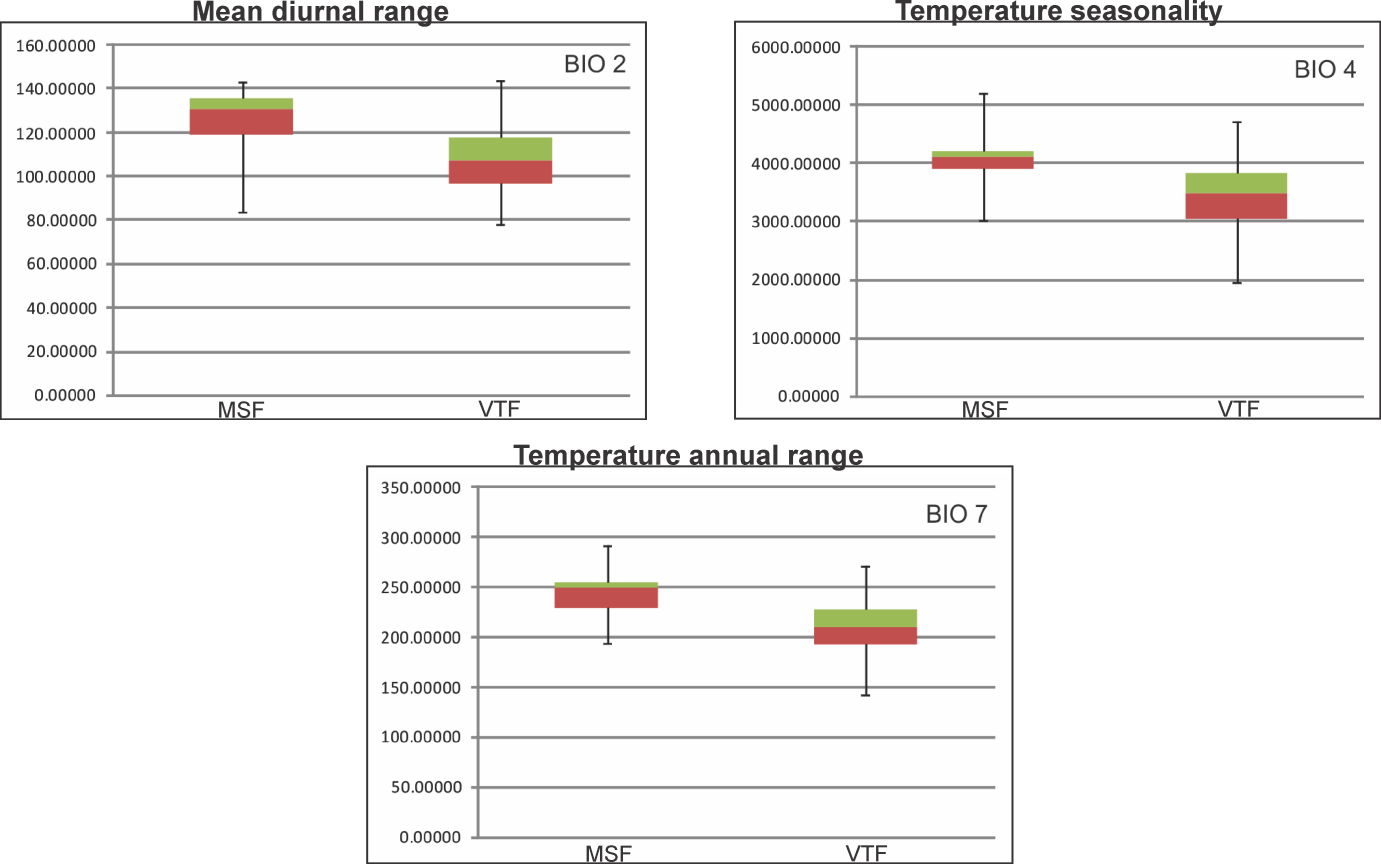
Boxplots of temperature related variables. Comparison of variables with high discrepancy for the Magellanic Subpolar Forest (MSF) and the Valdivian Temperate Forest (VTF) Late Pleistocene refugia (left MSF- right VTF). Boxes represent the upper (in green) and lower (in brown) 25% of the scores and are separated by the median. Units of the y-axes are in brackets beside chart headings.

The two assemblages that included enough species to identify Late Pleistocene refugia were MSF (8 spp.) and VTF (6 spp.). MSF refugia are located from approximately 37.5°/39°S and 71°/73°W to 52°/52.5°S and 71.5°/72°W, and VTF refugia are located between approximately 37°/42°S and 71°/74°W, both in Chile. MSF and VTF refugia are adjacent, with very little overlap (Fig 1B). The Kruskal- Wallis’s test between the MSF and VTF showed significant differences in all the environmental variables except for Isothermality, Mean Temperature of Driest Quarter and Mean Temperature of Warmest Quarter. Boxplots (Fig 2, S1 Figs) showed a climatic pattern during the Late Pleistocene similar to the present, with VTF being more humid than MSF.

**Fig 3:**
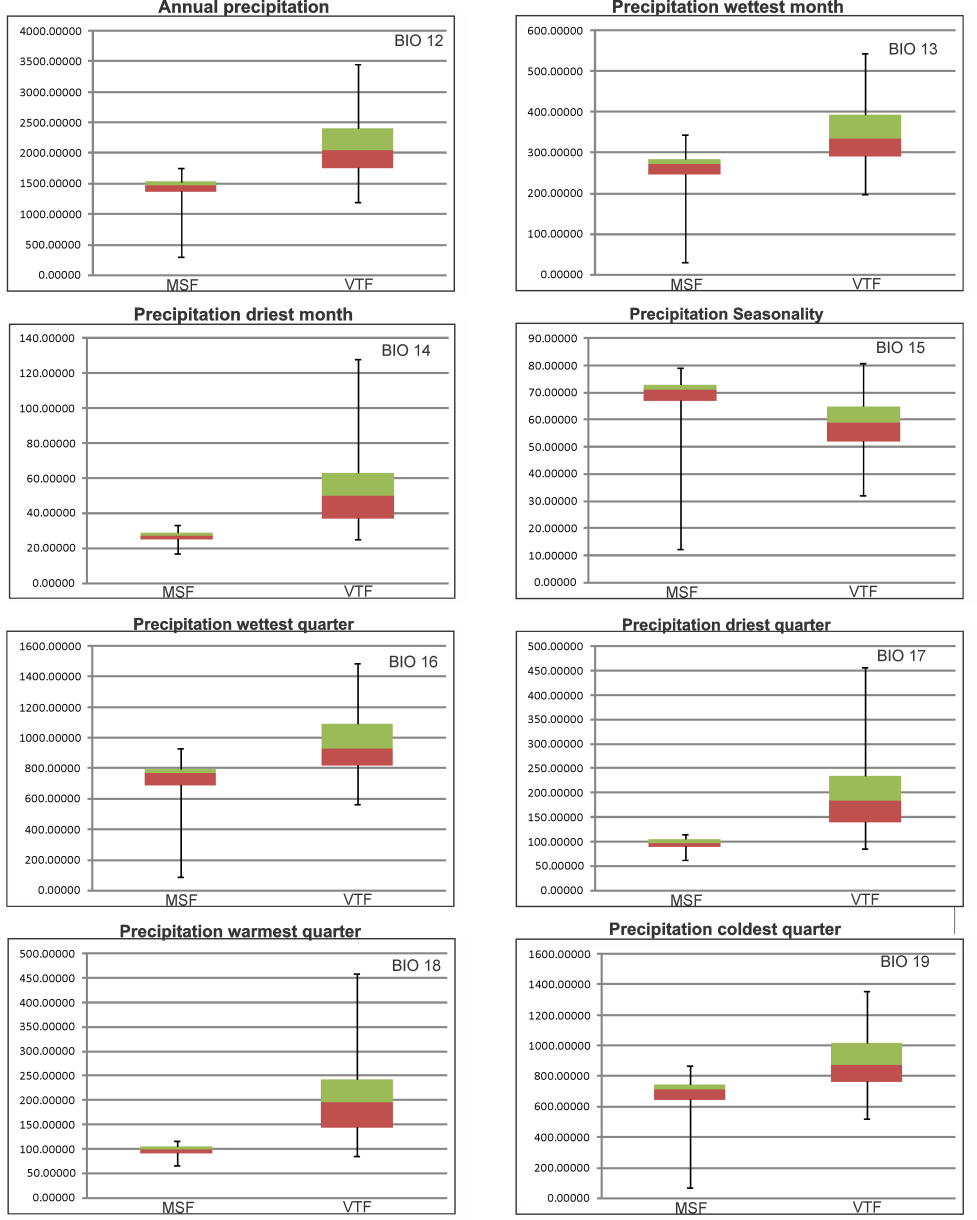
Boxplots of precipitation related variables. Comparison of variables with high discrepancy for the Magellanic Subpolar Forest (MSF) and the Valdivian Temperate Forest (VTF) Late Pleistocene refugia (left MSF- right VTF). Boxes represent the upper (in green) and lower (in brown) 25% of the scores and are separated by the median. Units of the y-axes are in brackets beside chart headings.

We identified eleven variables whose boxplots show a high discrepancy with no overlap of interquartiles (Figs 2 and 3). The variables related to temperature are: Mean Diurnal Range, Temperature Seasonality and Temperature Annual Range (Fig 2). The remaining eight variables are related to precipitation: Annual Precipitation, Precipitation of Wettest Month, Precipitation of Driest Month, Precipitation Seasonality, Precipitation of Wettest Quarter, Precipitation of Driest Quarter, Precipitation of Warmest Quarter and Precipitation of Coldest Quarter (Fig 3). There is one variable with partial overlap (Max Temperature of Warmest Month) and eight with complete overlap of interquartiles (Annual Mean Temperature, Isothermality, Min Temperature of Coldest Month, Mean Temperature of Wettest Quarter, Mean Temperature of Driest Quarter, Mean Temperature of Warmest Quarter and, Mean Temperature of Coldest Quarter) (S1 Figs).

### Insect-rich area maps (IAMs) and in situ refugia maps (IRMs)

**Fig 4:**
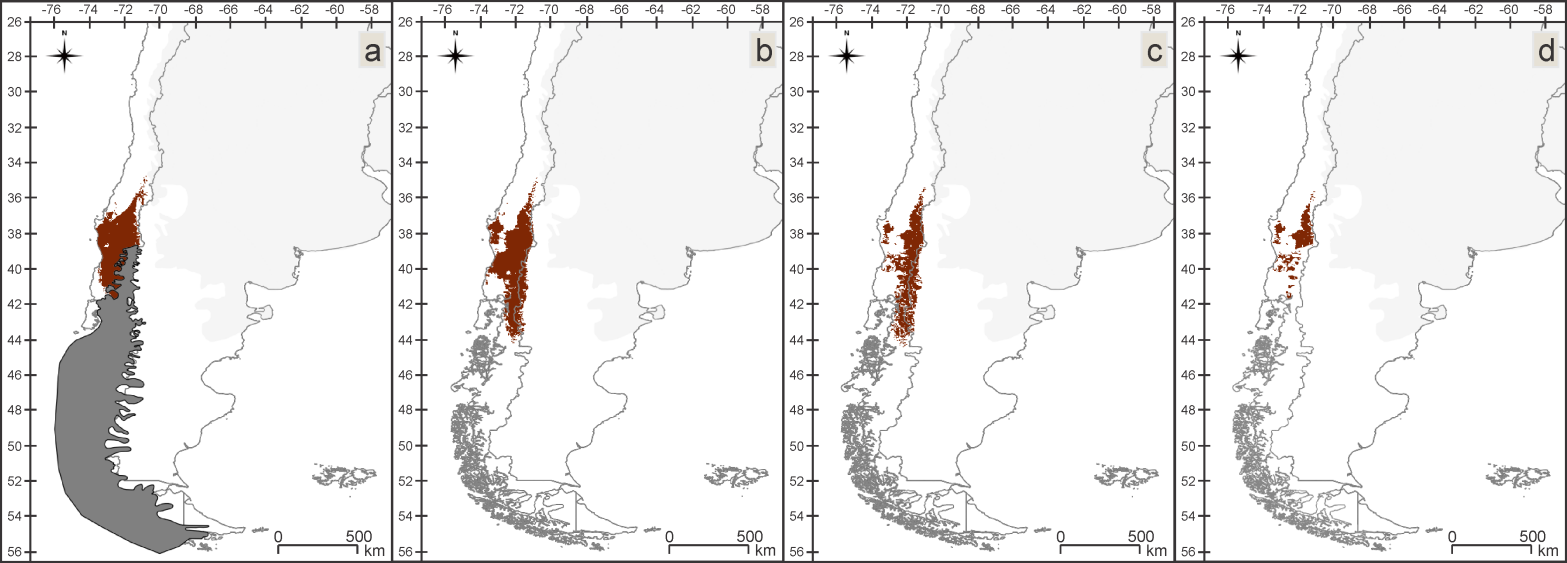
Valdivian Temperate Forest (VTF). Insect-rich area maps (IAMs), maps with areas where more than 80% of the species models matched (24 or more of the 31 species). (a) Late Pleistocene with the ice sheet. (b) Mid Holocene. (c) Present. (d) *in situ* refugia (IRM), map with the area where 80% or more of the species have found suitable conditions over all three time periods.

Between the Late Pleistocene and the Present, suitable areas for the VTF assemblage have undergone a process of fragmentation and retraction, losing territory towards west (Fig 4A–4C). During the Late Pleistocene a maximum of 29 species and during the Mid-Holocene and the Present a maximum of 30 out of 31 species were found matching per/pixel. The *in situ* refugia of this assemblage (Fig 4D) mostly include the northern areas of the IAMs for the three periods. Between the Late Pleistocene and the Mid Holocene and between the Mid Holocene and the Present the lost areas are similar.

**Fig 5:**
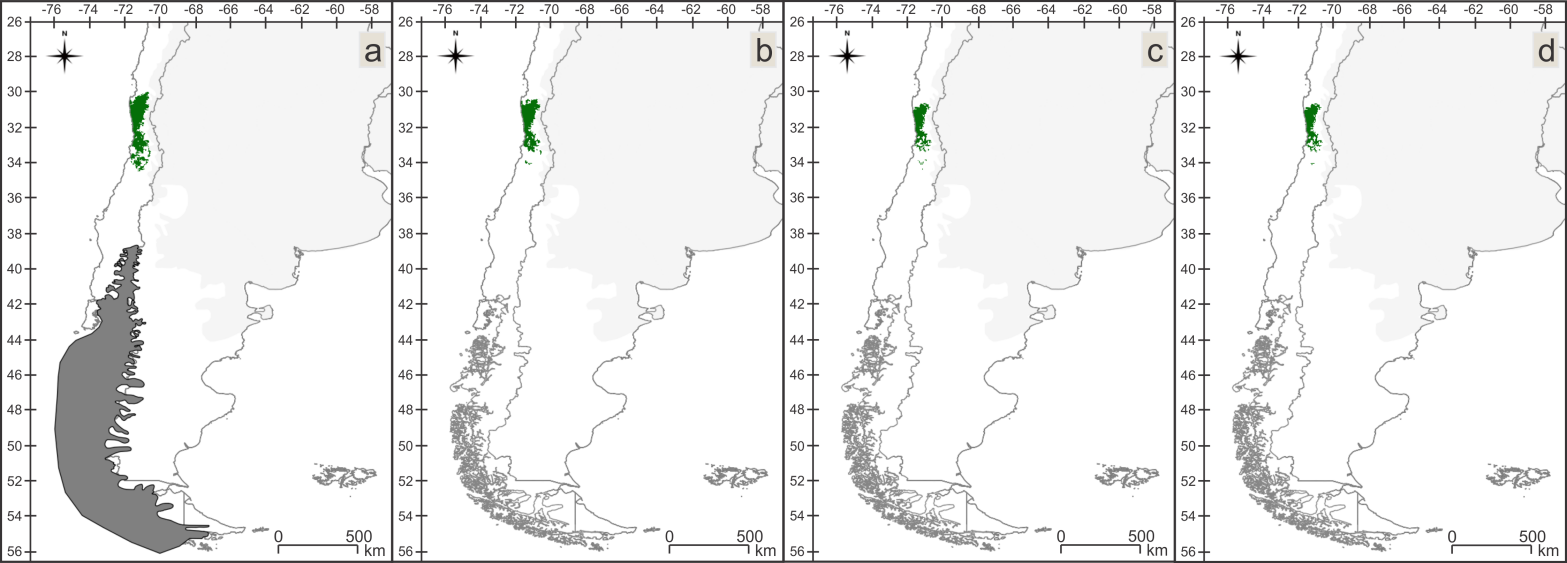
Chilean Matorral (CM). Insect-rich area maps (IAMs), maps with areas where more than 80% of the species models matched (8 or more of the 10 species). (a) Late Pleistocene with the ice sheet. (b) Mid Holocene. (c) Present. (d) *in situ* refugia (IRM), map with the area where 80% or more of the species have found suitable conditions over all three time periods.

In the CM assemblage, the suitable areas have suffered fewer modifications than in the other assemblages, including a small northern and southern retraction of the area to the Present (Fig 5A–5C). For the three periods, there are areas where all the species of the assemblage match. The *in situ* refugia of the CM assemblage (Fig 5D) has a similar pattern to the Present IAM. From the Late Pleistocene to the Mid Holocene 30% of the area was lost, and between the Mid Holocene and the Present 27%.

**Fig 6:**
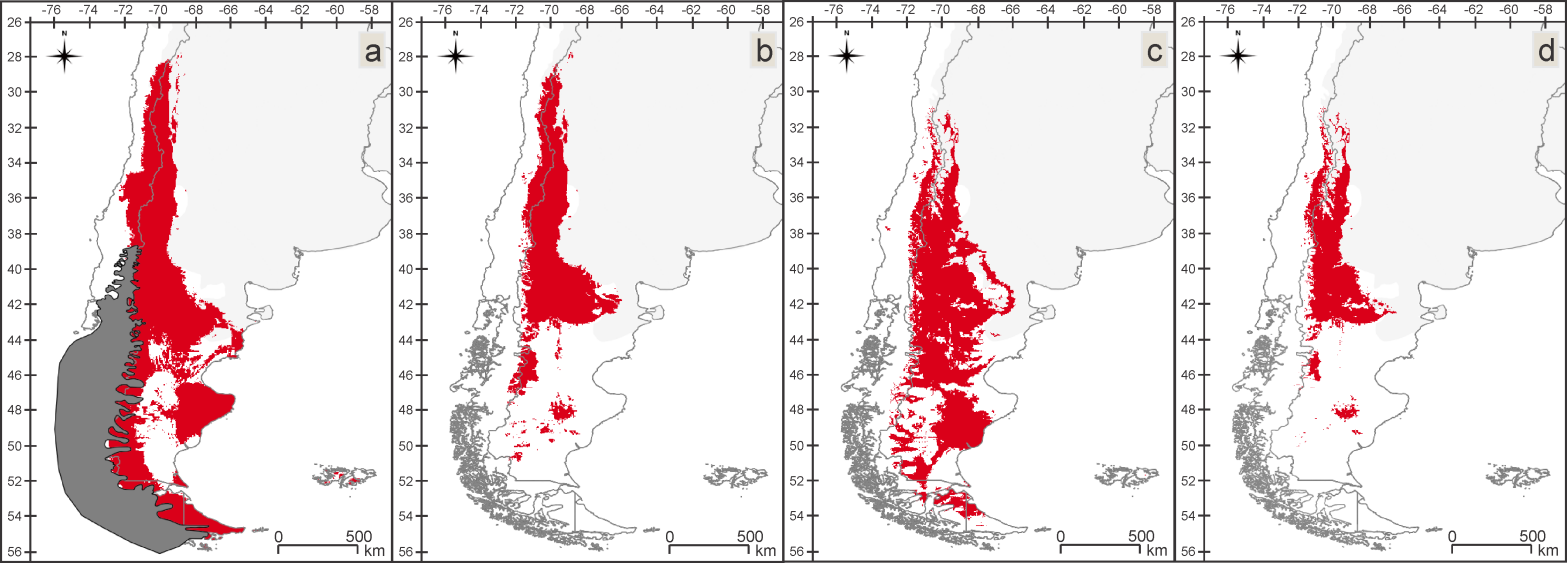
Patagonian Steppe (PS). Insect-rich area maps (IAMs), maps with areas where more than 80% of the species models matched (6 or more of the 8 species). (a) Late Pleistocene with the ice sheet. (b) Mid Holocene. (c) Present. (d) *in situ* refugia (IRM), map with the area where 80% or more of the species have found suitable conditions over all three time periods.

For the PS assemblage, we found a narrower, more fragmented area for the Present than for the Late Pleistocene, with loss of territory in the north and south (Fig 6A–6C). For all three periods, we recovered regions where all the species in the assemblage match. The PS assemblage *in situ* refugia (Fig 6D) follows a similar pattern to the northern part of the Present IAM. Major loss of areas occurred from the Late Pleistocene to the Mid Holocene (approximately 80%).

**Fig 7:**
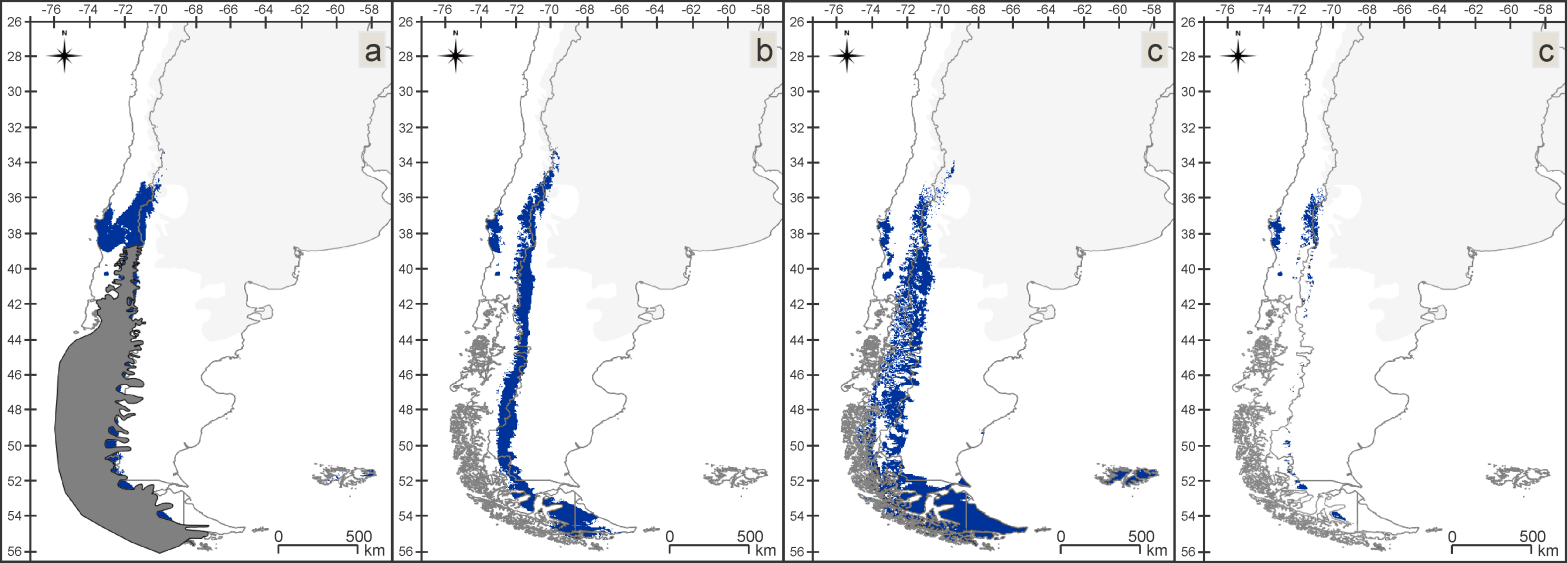
Magellanic Subpolar Forest (MSF). Insect-rich area maps (IAMs), maps with areas where more than 80% of the species models matched (8 or more of the 10 species). (a) Late Pleistocene with the ice sheet. (b) Mid Holocene. (c) Present. (d) *in situ* refugia (IRM), map with the area where 80% or more of the species have found suitable conditions over all three time periods.

The MSF assemblage is larger and more fragmented today than it was during the Late Pleistocene (Fig 7A–7C). In the Present, suitable areas have expanded to the south, reaching the continental limits. In the suitable areas of all three periods, we found regions where all the species in the assemblage match. The *in situ* refugia of the MSF (Fig 7D) is by far the smallest area as it is the ecoregion which was most affected by the Late Pleistocene ice sheet. The areas lost from the Mid Holocene to the Present are insignificant, representing approximately 6% of the lost areas.

### Consistency analysis of *in situ* refugia, Pleistocene refugia and endemic species

**Fig 8:**
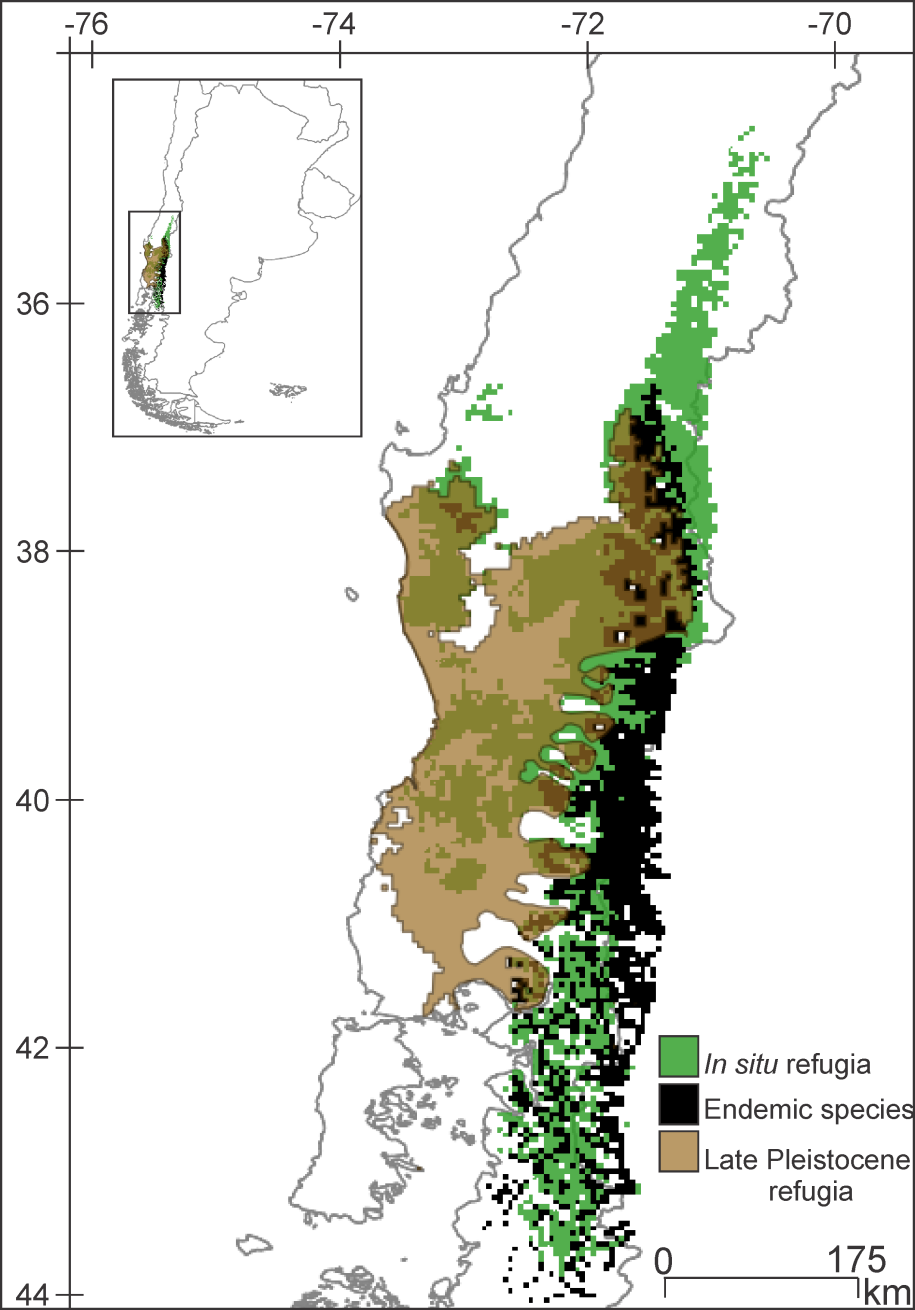
Congruence of *in situ* refugia, Pleistocene refugia and endemic species distributions of the VTF. *In situ* refugia green; Late Pleistocene refugia light brown, and endemic species distributions black; areas where all three agree in brown.

The VTF assemblage consists of 31 species. The IRM was built with 22 of them, the PRM with six, and the ESM with the nine species endemic to the ecoregion (the PRM and ESM share only one species). All three maps show a congruent pattern (Fig 8). Moreover, the endemic species have highly congruent present potential distributions, as there is a large area where more than 80% of the species overlap.

## Discussion

Of the 68 species initially considered, we discarded nine; two because the models were not validated, one because it had broad niche breadth and six because we did not recover suitable climatic areas in the past. This could be explained by the fact that during the LGM, the sea level was approximately 120–140 m below the present level, and the Patagonian continental boundary was ca. 450 km away from its present location [35]. The area where these species could have found suitable conditions was therefore not considered by the models.

Our results indicate that since the Late Pleistocene there has been a loss of potentially suitable territory for all the insect groups studied, for most of the endemic species, and for all the species assemblages except MSF. In general terms it would seem that most of these species would have had broader distributions when climate was colder. Among the ecoregions considered, MSF is probably the one where temperatures have remained coldest because of its more austral location. Thus, most of its species are not undergoing a process of retraction, in contrast to the species in the other ecoregions.

Ecoregions have been identified on the basis of a large amount of evidence from different sources [19]. Our results indicate that insect distributions fit well within these area concepts. Evidence of this is the fact that we recovered large areas where most of the species in each assemblage share similar climatic niches (IAM), but with little overlap between the areas of the different assemblages. This suggests not only consistency in the climatic requirements of the assemblages but also that there are differences among them.

The identified Late Pleistocene refugia also show a similar pattern; even though VTF and MSF refugia are adjacent, there is very little overlap between them. When characterizing the climate of these refugia by comparison of boxplots, we found a distinct pattern where the precipitation variables were characteristic of each refuge and clearly different from each other. Moreover, these refugia are congruent with others identified for different taxa (32 terrestrial vertebrates and 20 plants) and with a different methodology, phylogeography [36], located approximately between 38.5°/ 41° S and -71°/-74° W. The refugia we have identified are also fairly consistent with the populations of *Nothofagus* Blume that were recognized as having an ancient genetic pattern [37]. Suggestively, among the species used for this study as evidence to identify refugia, such as the terrestrial Hemiptera, Coleoptera and Diptera, several are ecologically related to *Nothofagus* forests [38–40]. The fossil evidence found of a Coleoptera, Curculionidae (*Listroderes dentipennis* Germain) in the Puerto Octay locality (40°58'27.876" S; 72°53'0.25" W) is also consistent with the refugia recovered herein, as it would have inhabited moorland and forested areas during the Late Pleistocene [10].

It is accepted that areas with long-term climate stability have a key role in promoting persistence of biodiversity, especially when regional or global climate conditions change [4]. Areas with high climatic stability have local factors that buffer the surrounding regional climate change and therefore allow the survival of ancient lineages and the preservation of the genetic variation of the persisting populations [41]. These areas are also important for increasing the probabilities of survival of new clades without reuniting with other clades [4]. The *in situ* refugia we recovered currently house 88% of the study species, and eight of the 13 species endemic to the ecoregions, therefore they harbor a high number of endemic species and great diversity. Furthermore, the potential distributions of most of these species (63%) are currently retracting, and consequently, in the context of current global warming, the distributions of these species will continue with this tendency.

The high biodiversity and endemicity of the stable areas can reflect their ancient history as safeguard areas. Thus, *in situ* refugia may play an important role in protecting current biodiversity by acting as buffers against the impacts of accelerated global warming in the future [4, 42]. Our *in situ* refugia are consistent with areas where climate will remain stable enough for the survival of fourteen insect species under future scenarios of global warming for 2050 [43]. Therefore, *in situ* refugia are key areas to consider when strategies are planned for the conservation of areas that are important in terms of biodiversity.

We consider that the high consistency between the present potential distribution of the endemic species, the Late Pleistocene refugia and the *in situ* refugia suggests that the methodology presented herein is very useful for determining environmentally meaningful areas of high conservational importance.

The proposed methodology can be applied–considering a series of assumptions–to different groups of organisms and regions, for the recognition of climatically suitable areas for hosting a high number of species, climatically stable areas over time and refugia. A similar approach considering other/more endemic biota and ecoregions would help to further understand the effect of past and future climate change on the biota of the Andean region and its current biodiversity patterns.

## Supporting information

S1 Table Full dataset. Distributional records of the species studied.
(PDF)

S1 Figs Boxplots. Comparison of variables with low discrepancy for the Magellanic Subpolar Forest (MSF) and the Valdivian Temperate Forest (VTF) Late Pleistocene refugia.
(PDF)
