# Supplementary material for: The fate of endemic insects of the Andean region under the effect of global warming
Source: PLoS One. 2017 Oct 16;12(10):e0186655. doi: 10.1371/journal.pone.0186655 (PMC5643147; doi:10.1371/journal.pone.0186655)
Supplement: S1 Table — Distributional records of the species studied. (PDF) [file pone.0186655.s001.pdf]

Plos One

A PROCEDURE TO ELUCIDATE THE FATE OF ENDEMIC INSECTS OF THE ANDEAN REGION UNDER THE EFFECT OF GLOBAL WARMING.

Montemayor S.I.; Melo M.C.; Scattolini M.C.; Pocco M.E.; del Río M.G.; Dellapé G.; Scheibler E.E.;
Roig S.A.; Cazorla C.G.; Dellapé P.M.

Order	Species	Lon_ext	Lat_ext	Training AUC	P_value	Number of records before Moran	After Moran
Coleoptera	*Dasydema_hyrtella* Blanchard	-70.6666	-33.4500	0, 9799	0.0014	23	9
Coleoptera	*Dasydema_hyrtella* Blanchard	-73.6666	-42.3833	0, 9799	0.0014	23	9
Coleoptera	*Dasydema_hyrtella* Blanchard	-74.0333	-42.0500	0, 9799	0.0014	23	9
Coleoptera	*Dasydema_hyrtella* Blanchard	-67.6666	-55.0833	0, 9799	0.0014	23	9
Coleoptera	*Dasydema_hyrtella* Blanchard	-70.9166	-53.1500	0, 9799	0.0014	23	9
Coleoptera	*Dasydema_hyrtella* Blanchard	-72.9166	-51.6666	0, 9799	0.0014	23	9
Coleoptera	*Dasydema_hyrtella* Blanchard	-72.1166	-52.5333	0, 9799	0.0014	23	9
Coleoptera	*Dasydema_hyrtella* Blanchard	-71.5000	-53.0000	0, 9799	0.0014	23	9
Coleoptera	*Dasydema_hyrtella* Blanchard	-66.9500	-55.0333	0, 9799	0.0014	23	9
Coleoptera	*Dasydema_hyrtella* Blanchard	-73.9333	-38.3666	0, 9799	0.0014	23	9
Coleoptera	*Dasydema_hyrtella* Blanchard	-72.2166	-39.2666	0, 9799	0.0014	23	9
Coleoptera	*Dasydema_hyrtella* Blanchard	-72.9369	-41.4716	0, 9799	0.0014	23	9
Coleoptera	*Dasydema_hyrtella* Blanchard	-72.6333	-41.4333	0, 9799	0.0014	23	9
Coleoptera	*Dasydema_hyrtella* Blanchard	-72.9833	-41.3166	0, 9799	0.0014	23	9
Coleoptera	*Dasydema_hyrtella* Blanchard	-71.6333	-38.6000	0, 9799	0.0014	23	9
Coleoptera	*Dasydema_hyrtella* Blanchard	-73.7166	-40.4666	0, 9799	0.0014	23	9
Coleoptera	*Hybreoleptops_roseus* Elgueta	-71.95000	-34.20000	0.9991	0	15	13
Coleoptera	*Hybreoleptops_roseus* Elgueta	-71.90000	-34.96660	0.9991	0	15	13
Coleoptera	*Hybreoleptops_roseus* Elgueta	-72.66660	-35.88330	0.9991	0	15	13
Coleoptera	*Hybreoleptops_roseus* Elgueta	-72.69270	-35.98330	0.9991	0	15	13
Coleoptera	*Hybreoleptops_roseus* Elgueta	-72.78330	-36.13330	0.9991	0	15	13
Coleoptera	*Hybreoleptops_roseus* Elgueta	-72.35000	-35.60000	0.9991	0	15	13
Coleoptera	*Hybreoleptops_roseus* Elgueta	-72.35000	-35.61660	0.9991	0	15	13
Coleoptera	*Hybreoleptops_roseus* Elgueta	-72.35000	-35.96660	0.9991	0	15	13
Coleoptera	*Hybreoleptops_roseus* Elgueta	-72.25000	-35.30000	0.9991	0	15	13
Coleoptera	*Hybreoleptops_roseus* Elgueta	-72.08330	-35.11660	0.9991	0	15	13
Coleoptera	*Hybreoleptops_roseus* Elgueta	-72.25000	-35.25000	0.9991	0	15	13
Coleoptera	*Hybreoleptops_roseus* Elgueta	-72.21660	-35.25000	0.9991	0	15	13
Coleoptera	*Hybreoleptops_roseus* Elgueta	-72.68330	-35.90000	0.9991	0	15	13
Coleoptera	*Hybreoleptops_roseus* Elgueta	-72.00000	-34.38330	0.9991	0	15	13
Coleoptera	*Hybreoleptops_roseus* Elgueta	-72.63330	-35.83330	0.9991	0	15	13
Coleoptera	*Hybreoleptops tuberculifer* (Boheman)	-71.8666	-41.0500	0.9965	0	11	11
Coleoptera	*Hybreoleptops tuberculifer* (Boheman)	-72.9833	-41.3166	0.9965	0	11	11
Coleoptera	*Hybreoleptops tuberculifer* (Boheman)	-71.9666	-39.2666	0.9965	0	11	11

Coleoptera	*Hybreoleptops tuberculifer* (Boheman)	-73.7166	-40.4666	0.9965	0	11	11
Coleoptera	*Hybreoleptops tuberculifer* (Boheman)	-72.3666	-40.6833	0.9965	0	11	11
Coleoptera	*Hybreoleptops tuberculifer* (Boheman)	-73.1166	-39.7833	0.9965	0	11	11
Coleoptera	*Hybreoleptops tuberculifer* (Boheman)	-73.6666	-42.3833	0.9965	0	11	11
Coleoptera	*Hybreoleptops tuberculifer* (Boheman)	-71.7166	-40.1500	0.9965	0	11	11
Coleoptera	*Hybreoleptops tuberculifer* (Boheman)	-71.3000	-41.1500	0.9965	0	11	11
Coleoptera	*Hybreoleptops tuberculifer* (Boheman)	-71.5500	-40.9333	0.9965	0	11	11
Coleoptera	*Hybreoleptops tuberculifer* (Boheman)	-71.5333	-41.3500	0.9965	0	11	11
Coleoptera	*Megalometis spinifer* Boheman	-73.7166	-40.4666	0.9975	0	11	9
Coleoptera	*Megalometis spinifer* Boheman	-72.5000	-40.6666	0.9975	0	11	9
Coleoptera	*Megalometis spinifer* Boheman	-73.8202	-41.8697	0.9975	0	11	9
Coleoptera	*Megalometis spinifer* Boheman	-73.4500	-42.4833	0.9975	0	11	9
Coleoptera	*Megalometis spinifer* Boheman	-74.0333	-42.0500	0.9975	0	11	9
Coleoptera	*Megalometis spinifer* Boheman	-73.7666	-42.4833	0.9975	0	11	9
Coleoptera	*Megalometis spinifer* Boheman	-73.6666	-42.3833	0.9975	0	11	9
Coleoptera	*Megalometis spinifer* Boheman	-72.3500	-39.6333	0.9975	0	11	9
Coleoptera	*Megalometis spinifer* Boheman	-73.6166	-41.0500	0.9975	0	11	9
Coleoptera	*Megalometis spinifer* Boheman	-73.6833	-41.6666	0.9975	0	11	9
Coleoptera	*Megalometis spinifer* Boheman	-73.0500	-36.8333	0.9975	0	11	9
Diptera	*Stilobezzia (Acanthohelea) patagonica* Ingram & Macfie	-71.3106	-41.1325	0.996	0	13	13
Diptera	*Stilobezzia (Acanthohelea) patagonica* Ingram & Macfie	-71.35	-40.1667	0.996	0	13	13
Diptera	*Stilobezzia (Acanthohelea) patagonica* Ingram & Macfie	-71.6667	-40.7833	0.996	0	13	13
Diptera	*Stilobezzia (Acanthohelea) patagonica* Ingram & Macfie	-71.4347	-40.1728	0.996	0	13	13
Diptera	*Stilobezzia (Acanthohelea) patagonica* Ingram & Macfie	-71.95	-40.2167	0.996	0	13	13
Diptera	*Stilobezzia (Acanthohelea) patagonica* Ingram & Macfie	-71.4344	-40.0408	0.996	0	13	13
Diptera	*Stilobezzia (Acanthohelea) patagonica* Ingram & Macfie	-71.2667	-40.2333	0.996	0	13	13
Diptera	*Stilobezzia (Acanthohelea) patagonica* Ingram & Macfie	-71.8833	-41.5833	0.996	0	13	13
Diptera	*Stilobezzia (Acanthohelea) patagonica* Ingram & Macfie	-71.05	-41.25	0.996	0	13	13
Diptera	*Stilobezzia (Acanthohelea) patagonica* Ingram & Macfie	-71.95	-41.85	0.996	0	13	13
Diptera	*Stilobezzia (Acanthohelea) patagonica* Ingram & Macfie	-72.6667	-36.4167	0.996	0	13	13
Diptera	*Stilobezzia (Acanthohelea) patagonica* Ingram & Macfie	-71.7333	-36.8	0.996	0	13	13
Diptera	*Stilobezzia (Acanthohelea) patagonica* Ingram & Macfie	-73.25	-39.8	0.996	0	13	13
Diptera	*Stilobezzia (Acanthohelea) borkenti* Cazorla & Spinelli	-71.3106	-41.1325	0.998	0	11	11
Diptera	*Stilobezzia (Acanthohelea) borkenti* Cazorla & Spinelli	-71.7075	-41.3544	0.998	0	11	11
Diptera	*Stilobezzia (Acanthohelea) borkenti* Cazorla & Spinelli	-71.2833	-41.1333	0.998	0	11	11
Diptera	*Stilobezzia (Acanthohelea) borkenti* Cazorla & Spinelli	-71.6069	-41.6017	0.998	0	11	11
Diptera	*Stilobezzia (Acanthohelea) borkenti* Cazorla & Spinelli	-71.4344	-40.0408	0.998	0	11	11
Diptera	*Stilobezzia (Acanthohelea) borkenti* Cazorla & Spinelli	-71.6722	-40.0995	0.998	0	11	11
Diptera	*Stilobezzia (Acanthohelea) borkenti* Cazorla & Spinelli	-71.6217	-40.8517	0.998	0	11	11
Diptera	*Stilobezzia (Acanthohelea) borkenti* Cazorla & Spinelli	-71.2925	-40.6897	0.998	0	11	11
Diptera	*Stilobezzia (Acanthohelea) borkenti* Cazorla & Spinelli	-71.0167	-39.9167	0.998	0	11	11

Diptera	*Stilobezzia (Acanthohelea) borkenti* Cazorla & Spinelli	-71.1908	-39.2386	0.998	0	11	11
Diptera	*Stilobezzia (Acanthohelea) borkenti* Cazorla & Spinelli	-72.3161	-40.6333	0.998	0	11	11
Diptera	*Stilobezzia (Acanthohelea) varia* Ingram & Macfie	-71.3106	-41.1322	0.993	0	33	19
Diptera	*Stilobezzia (Acanthohelea) varia* Ingram & Macfie	-72.1667	-41.8833	0.993	0	33	19
Diptera	*Stilobezzia (Acanthohelea) varia* Ingram & Macfie	-71.8156	-41.0261	0.993	0	33	19
Diptera	*Stilobezzia (Acanthohelea) varia* Ingram & Macfie	-71.2992	-41.2214	0.993	0	33	19
Diptera	*Stilobezzia (Acanthohelea) varia* Ingram & Macfie	-71.4103	-41.1867	0.993	0	33	19
Diptera	*Stilobezzia (Acanthohelea) varia* Ingram & Macfie	-71.8875	-41.1608	0.993	0	33	19
Diptera	*Stilobezzia (Acanthohelea) varia* Ingram & Macfie	-71.7367	-41.2411	0.993	0	33	19
Diptera	*Stilobezzia (Acanthohelea) varia* Ingram & Macfie	-71.5156	-41.2703	0.993	0	33	19
Diptera	*Stilobezzia (Acanthohelea) varia* Ingram & Macfie	-71.8072	-41.0869	0.993	0	33	19
Diptera	*Stilobezzia (Acanthohelea) varia* Ingram & Macfie	-71.7075	-41.3544	0.993	0	33	19
Diptera	*Stilobezzia (Acanthohelea) varia* Ingram & Macfie	-71.8075	-41.0392	0.993	0	33	19
Diptera	*Stilobezzia (Acanthohelea) varia* Ingram & Macfie	-71.3278	-41.1978	0.993	0	33	19
Diptera	*Stilobezzia (Acanthohelea) varia* Ingram & Macfie	-71.8	-40.6333	0.993	0	33	19
Diptera	*Stilobezzia (Acanthohelea) varia* Ingram & Macfie	-71.35	-40.1667	0.993	0	33	19
Diptera	*Stilobezzia (Acanthohelea) varia* Ingram & Macfie	-71.6578	-40.7853	0.993	0	33	19
Diptera	*Stilobezzia (Acanthohelea) varia* Ingram & Macfie	-71.5875	-39.7236	0.993	0	33	19
Diptera	*Stilobezzia (Acanthohelea) varia* Ingram & Macfie	-71.1439	-39.8697	0.993	0	33	19
Diptera	*Stilobezzia (Acanthohelea) varia* Ingram & Macfie	-71.4297	-39.5381	0.993	0	33	19
Diptera	*Stilobezzia (Acanthohelea) varia* Ingram & Macfie	-73.8333	-41.8667	0.993	0	33	19
Diptera	*Stilobezzia (Acanthohelea) varia* Ingram & Macfie	-72.9853	-41.3194	0.993	0	33	19
Diptera	*Stilobezzia (Acanthohelea) varia* Ingram & Macfie	-71.6997	-38.5192	0.993	0	33	19
Diptera	*Stilobezzia (Acanthohelea) varia* Ingram & Macfie	-71.4569	-38.5778	0.993	0	33	19
Diptera	*Stilobezzia (Acanthohelea) varia* Ingram & Macfie	-71.6497	-38.6667	0.993	0	33	19
Diptera	*Stilobezzia (Acanthohelea) varia* Ingram & Macfie	-73.25	-39.8	0.993	0	33	19
Diptera	*Stilobezzia (Acanthohelea) varia* Ingram & Macfie	-73.1703	-40.5494	0.993	0	33	19
Diptera	*Stilobezzia (Acanthohelea) varia* Ingram & Macfie	-72.2333	-42.8167	0.993	0	33	19
Diptera	*Stilobezzia (Acanthohelea) varia* Ingram & Macfie	-68.5	-54.8333	0.993	0	33	19
Diptera	*Stilobezzia (Acanthohelea) varia* Ingram & Macfie	-67.25	-55.75	0.993	0	33	19
Diptera	*Stilobezzia (Acanthohelea) varia* Ingram & Macfie	-72.8022	-50.5261	0.993	0	33	19
Diptera	*Stilobezzia (Acanthohelea) varia* Ingram & Macfie	-72.8647	-49.0372	0.993	0	33	19
Diptera	*Stilobezzia (Acanthohelea) varia* Ingram & Macfie	-72.4164	-49.1661	0.993	0	33	19
Diptera	*Stilobezzia (Acanthohelea) varia* Ingram & Macfie	-71.715	-42.8144	0.993	0	33	19
Diptera	*Stilobezzia (Acanthohelea) varia* Ingram & Macfie	-72	-42.23	0.993	0	33	19
Diptera	*Stilobezzia (Acanthohelea) curvistyla* Cazorla & Spinelli	-71.85	-42.8	0.998	0	13	10
Diptera	*Stilobezzia (Acanthohelea) curvistyla* Cazorla & Spinelli	-71.4103	-41.1867	0.998	0	13	10
Diptera	*Stilobezzia (Acanthohelea) curvistyla* Cazorla & Spinelli	-71.8156	-41.0261	0.998	0	13	10
Diptera	*Stilobezzia (Acanthohelea) curvistyla* Cazorla & Spinelli	-71.7367	-41.2411	0.998	0	13	10
Diptera	*Stilobezzia (Acanthohelea) curvistyla* Cazorla & Spinelli	-71.8278	-41.0017	0.998	0	13	10
Diptera	*Stilobezzia (Acanthohelea) curvistyla* Cazorla & Spinelli	-71.5781	-40.8786	0.998	0	13	10

Diptera	*Stilobezzia (Acanthohelea) curvistyla* Cazorla & Spinelli	-71.4128	-40.6508	0.998	0	13	10
Diptera	*Stilobezzia (Acanthohelea) curvistyla* Cazorla & Spinelli	-71.95	-40.1667	0.998	0	13	10
Diptera	*Stilobezzia (Acanthohelea) curvistyla* Cazorla & Spinelli	-71.35	-40.1667	0.998	0	13	10
Diptera	*Stilobezzia (Acanthohelea) curvistyla* Cazorla & Spinelli	-72.5856	-41.1181	0.998	0	13	10
Diptera	*Stilobezzia (Acanthohelea) curvistyla* Cazorla & Spinelli	-72.5378	-41.2067	0.998	0	13	10
Diptera	*Stilobezzia (Acanthohelea) curvistyla* Cazorla & Spinelli	-72.3167	-40.6333	0.998	0	13	10
Diptera	*Stilobezzia (Acanthohelea) curvistyla* Cazorla & Spinelli	-73.8333	-41.8667	0.998	0	13	10
Diptera	*Stilobezzia (Acanthohelea) furva* Ingram & Macfie	-72	-42.8	0.995	0	20	20
Diptera	*Stilobezzia (Acanthohelea) furva* Ingram & Macfie	-71.4103	-41.1867	0.995	0	20	20
Diptera	*Stilobezzia (Acanthohelea) furva* Ingram & Macfie	-71.2992	-41.2214	0.995	0	20	20
Diptera	*Stilobezzia (Acanthohelea) furva* Ingram & Macfie	-71.3106	-41.1322	0.995	0	20	20
Diptera	*Stilobezzia (Acanthohelea) furva* Ingram & Macfie	-71.6578	-40.7853	0.995	0	20	20
Diptera	*Stilobezzia (Acanthohelea) furva* Ingram & Macfie	-71.6667	-40.7	0.995	0	20	20
Diptera	*Stilobezzia (Acanthohelea) furva* Ingram & Macfie	-71.8833	-41.5833	0.995	0	20	20
Diptera	*Stilobezzia (Acanthohelea) furva* Ingram & Macfie	-71.2667	-40.2333	0.995	0	20	20
Diptera	*Stilobezzia (Acanthohelea) furva* Ingram & Macfie	-71.5	-40.1833	0.995	0	20	20
Diptera	*Stilobezzia (Acanthohelea) furva* Ingram & Macfie	-71.7166	-40.15	0.995	0	20	20
Diptera	*Stilobezzia (Acanthohelea) furva* Ingram & Macfie	-71.4344	-40.0408	0.995	0	20	20
Diptera	*Stilobezzia (Acanthohelea) furva* Ingram & Macfie	-71.6722	-40.0994	0.995	0	20	20
Diptera	*Stilobezzia (Acanthohelea) furva* Ingram & Macfie	-71.58	-39.7133	0.995	0	20	20
Diptera	*Stilobezzia (Acanthohelea) furva* Ingram & Macfie	-72.5856	-41.1181	0.995	0	20	20
Diptera	*Stilobezzia (Acanthohelea) furva* Ingram & Macfie	-72.9667	-41.3	0.995	0	20	20
Diptera	*Stilobezzia (Acanthohelea) furva* Ingram & Macfie	-73.1703	-40.5494	0.995	0	20	20
Diptera	*Stilobezzia (Acanthohelea) furva* Ingram & Macfie	-71.9833	-39.7833	0.995	0	20	20
Diptera	*Stilobezzia (Acanthohelea) furva* Ingram & Macfie	-71.6997	-38.5192	0.995	0	20	20
Diptera	*Stilobezzia (Acanthohelea) furva* Ingram & Macfie	-71.7333	-36.8	0.995	0	20	20
Diptera	*Stilobezzia (Acanthohelea) furva* Ingram & Macfie	-70.45	-33.5833	0.995	0	20	20
Diptera	*Stilobezzia (Acanthohelea) bicinctipes* Ingram & Macfie	-71.5781	-40.8786	0.998	0	10	10
Diptera	*Stilobezzia (Acanthohelea) bicinctipes* Ingram & Macfie	-71.8156	-41.0261	0.998	0	10	10
Diptera	*Stilobezzia (Acanthohelea) bicinctipes* Ingram & Macfie	-72.9667	-41.3	0.998	0	10	10
Diptera	*Stilobezzia (Acanthohelea) bicinctipes* Ingram & Macfie	-71.8667	-41.05	0.998	0	10	10
Diptera	*Stilobezzia (Acanthohelea) bicinctipes* Ingram & Macfie	-72.8	-40.4667	0.998	0	10	10
Diptera	*Stilobezzia (Acanthohelea) bicinctipes* Ingram & Macfie	-73.1703	-40.5494	0.998	0	10	10
Diptera	*Stilobezzia (Acanthohelea) bicinctipes* Ingram & Macfie	-73.25	-39.8	0.998	0	10	10
Diptera	*Stilobezzia (Acanthohelea) bicinctipes* Ingram & Macfie	-72.3167	-40.6333	0.998	0	10	10
Diptera	*Stilobezzia (Acanthohelea) bicinctipes* Ingram & Macfie	-72.2333	-42.8167	0.998	0	10	10
Diptera	*Stilobezzia (Acanthohelea) bicinctipes* Ingram & Macfie	-72.5856	-41.1181	0.998	0	10	10
Diptera	*Forcipomyia (Forcipomyia) multipicta* Ingram & Macfie	-70.7333	-40.1167	0.991	0	28	21
Diptera	*Forcipomyia (Forcipomyia) multipicta* Ingram & Macfie	-71.65	-40.15	0.991	0	28	21
Diptera	*Forcipomyia (Forcipomyia) multipicta* Ingram & Macfie	-71.1439	-39.8697	0.991	0	28	21
Diptera	*Forcipomyia (Forcipomyia) multipicta* Ingram & Macfie	-71.6667	-41.7167	0.991	0	28	21

Diptera	*Forcipomyia (Forcipomyia) multipicta* Ingram & Macfie	-71.8833	-41.5833	0.991	0	28	21
Diptera	*Forcipomyia (Forcipomyia) multipicta* Ingram & Macfie	-71.3106	-41.1322	0.991	0	28	21
Diptera	*Forcipomyia (Forcipomyia) multipicta* Ingram & Macfie	-71.05	-41.25	0.991	0	28	21
Diptera	*Forcipomyia (Forcipomyia) multipicta* Ingram & Macfie	-71.8181	-41.0217	0.991	0	28	21
Diptera	*Forcipomyia (Forcipomyia) multipicta* Ingram & Macfie	-71.5156	-41.2703	0.991	0	28	21
Diptera	*Forcipomyia (Forcipomyia) multipicta* Ingram & Macfie	-71.8292	-41.0156	0.991	0	28	21
Diptera	*Forcipomyia (Forcipomyia) multipicta* Ingram & Macfie	-71.8072	-41.0869	0.991	0	28	21
Diptera	*Forcipomyia (Forcipomyia) multipicta* Ingram & Macfie	-71.8153	-41.0261	0.991	0	28	21
Diptera	*Forcipomyia (Forcipomyia) multipicta* Ingram & Macfie	-71.8075	-41.0392	0.991	0	28	21
Diptera	*Forcipomyia (Forcipomyia) multipicta* Ingram & Macfie	-71.7828	-41.2356	0.991	0	28	21
Diptera	*Forcipomyia (Forcipomyia) multipicta* Ingram & Macfie	-71.4125	-40.6508	0.991	0	28	21
Diptera	*Forcipomyia (Forcipomyia) multipicta* Ingram & Macfie	-71.5781	-40.8786	0.991	0	28	21
Diptera	*Forcipomyia (Forcipomyia) multipicta* Ingram & Macfie	-71.6122	-40.48	0.991	0	28	21
Diptera	*Forcipomyia (Forcipomyia) multipicta* Ingram & Macfie	-71.7547	-41.3358	0.991	0	28	21
Diptera	*Forcipomyia (Forcipomyia) multipicta* Ingram & Macfie	-72	-42.38	0.991	0	28	21
Diptera	*Forcipomyia (Forcipomyia) multipicta* Ingram & Macfie	-71.5322	-40.1381	0.991	0	28	21
Diptera	*Forcipomyia (Forcipomyia) multipicta* Ingram & Macfie	-72.8647	-49.0372	0.991	0	28	21
Diptera	*Forcipomyia (Forcipomyia) multipicta* Ingram & Macfie	-72.8647	-49.1661	0.991	0	28	21
Diptera	*Forcipomyia (Forcipomyia) multipicta* Ingram & Macfie	-68.3472	-53.9214	0.991	0	28	21
Diptera	*Forcipomyia (Forcipomyia) multipicta* Ingram & Macfie	-66.9578	-54.9267	0.991	0	28	21
Diptera	*Forcipomyia (Forcipomyia) multipicta* Ingram & Macfie	-68.5936	-54.8564	0.991	0	28	21
Diptera	*Forcipomyia (Forcipomyia) multipicta* Ingram & Macfie	-73.1703	-40.5494	0.991	0	28	21
Diptera	*Forcipomyia (Forcipomyia) multipicta* Ingram & Macfie	-71.8753	-41.0486	0.991	0	28	21
Diptera	*Forcipomyia (Forcipomyia) multipicta* Ingram & Macfie	-67.1419	-55.8614	0.991	0	28	21
Diptera	*Forcipomyia (Forcipomyia) chilensis* (Philippi)	-70.6667	-33.45	0.986	0	9	9
Diptera	*Forcipomyia (Forcipomyia) chilensis* (Philippi)	-72.6667	-36.4167	0.986	0	9	9
Diptera	*Forcipomyia (Forcipomyia) chilensis* (Philippi)	-71.6667	-40.1167	0.986	0	9	9
Diptera	*Forcipomyia (Forcipomyia) chilensis* (Philippi)	-71.1439	-39.8697	0.986	0	9	9
Diptera	*Forcipomyia (Forcipomyia) chilensis* (Philippi)	-70.7333	-40.1167	0.986	0	9	9
Diptera	*Forcipomyia (Forcipomyia) chilensis* (Philippi)	-71.6667	-41.7167	0.986	0	9	9
Diptera	*Forcipomyia (Forcipomyia) chilensis* (Philippi)	-72.4164	-49.1661	0.986	0	9	9
Diptera	*Forcipomyia (Forcipomyia) chilensis* (Philippi)	-68.5728	-54.8556	0.986	0	9	9
Diptera	*Forcipomyia (Forcipomyia) chilensis* (Philippi)	-70.9833	-37.85	0.986	0	9	9
Diptera	*Forcipomyia (Forcipomyia) fusca* (Philippi)	-70.6608	-30.7467	0.969	0	15	15
Diptera	*Forcipomyia (Forcipomyia) fusca* (Philippi)	-71.3106	-41.135	0.969	0	15	15
Diptera	*Forcipomyia (Forcipomyia) fusca* (Philippi)	-71.35	-40.1667	0.969	0	15	15
Diptera	*Forcipomyia (Forcipomyia) fusca* (Philippi)	-71.1439	-39.8697	0.969	0	15	15
Diptera	*Forcipomyia (Forcipomyia) fusca* (Philippi)	-71.2983	-40.3597	0.969	0	15	15
Diptera	*Forcipomyia (Forcipomyia) fusca* (Philippi)	-71.7166	-40.15	0.969	0	15	15
Diptera	*Forcipomyia (Forcipomyia) fusca* (Philippi)	-71.4103	-41.1867	0.969	0	15	15
Diptera	*Forcipomyia (Forcipomyia) fusca* (Philippi)	-71.8833	-41.7667	0.969	0	15	15

Diptera	*Forcipomyia (Forcipomyia) fusca* (Philippi)	-71.8833	-41.5833	0.969	0	15	15
Diptera	*Forcipomyia (Forcipomyia) fusca* (Philippi)	-71.85	-42.6667	0.969	0	15	15
Diptera	*Forcipomyia (Forcipomyia) fusca* (Philippi)	-72.8647	-49.0372	0.969	0	15	15
Diptera	*Forcipomyia (Forcipomyia) fusca* (Philippi)	-68.5728	-54.8556	0.969	0	15	15
Diptera	*Forcipomyia (Forcipomyia) fusca* (Philippi)	-71.7333	-36.8	0.969	0	15	15
Diptera	*Forcipomyia (Forcipomyia) fusca* (Philippi)	-71.6667	-30.6667	0.969	0	15	15
Diptera	*Forcipomyia (Forcipomyia) fusca* (Philippi)	-67.1419	-55.8614	0.969	0	15	15
Diptera	*Forcipomyia (Euprojoannisia) setosicrus* (Kieffer)	-72.7667	-36.6167	0.966	0	11	11
Diptera	*Forcipomyia (Euprojoannisia) setosicrus* (Kieffer)	-73.0333	-36.7	0.966	0	11	11
Diptera	*Forcipomyia (Euprojoannisia) setosicrus* (Kieffer)	-72.4833	-40.2833	0.966	0	11	11
Diptera	*Forcipomyia (Euprojoannisia) setosicrus* (Kieffer)	-71.1908	-39.2386	0.966	0	11	11
Diptera	*Forcipomyia (Euprojoannisia) setosicrus* (Kieffer)	-71.2833	-38.9	0.966	0	11	11
Diptera	*Forcipomyia (Euprojoannisia) setosicrus* (Kieffer)	-72.1	-41.7333	0.966	0	11	11
Diptera	*Forcipomyia (Euprojoannisia) setosicrus* (Kieffer)	-71.8156	-41.0261	0.966	0	11	11
Diptera	*Forcipomyia (Euprojoannisia) setosicrus* (Kieffer)	-71.8833	-41.5833	0.966	0	11	11
Diptera	*Forcipomyia (Euprojoannisia) setosicrus* (Kieffer)	-72	-42.38	0.966	0	11	11
Diptera	*Forcipomyia (Euprojoannisia) setosicrus* (Kieffer)	-71.8333	-44.8833	0.966	0	11	11
Diptera	*Forcipomyia (Euprojoannisia) setosicrus* (Kieffer)	-68.5728	-54.8556	0.966	0	11	11
Diptera	*Atrichopogon obnubilus* Ingram & Macfie	-71.3106	-41.1325	0.994	0	13	13
Diptera	*Atrichopogon obnubilus* Ingram & Macfie	-71.6722	-41.094	0.994	0	13	13
Diptera	*Atrichopogon obnubilus* Ingram & Macfie	-71.1331	-38.9167	0.994	0	13	13
Diptera	*Atrichopogon obnubilus* Ingram & Macfie	-71.0296	-36.8447	0.994	0	13	13
Diptera	*Atrichopogon obnubilus* Ingram & Macfie	-71.305	-39.4194	0.994	0	13	13
Diptera	*Atrichopogon obnubilus* Ingram & Macfie	-71.05	-41.25	0.994	0	13	13
Diptera	*Atrichopogon obnubilus* Ingram & Macfie	-71.5167	-41.9667	0.994	0	13	13
Diptera	*Atrichopogon obnubilus* Ingram & Macfie	-72	-42.32	0.994	0	13	13
Diptera	*Atrichopogon obnubilus* Ingram & Macfie	-72.8647	-49.0372	0.994	0	13	13
Diptera	*Atrichopogon obnubilus* Ingram & Macfie	-72.4164	-49.1661	0.994	0	13	13
Diptera	*Atrichopogon obnubilus* Ingram & Macfie	-72.9667	-41.3	0.994	0	13	13
Diptera	*Atrichopogon obnubilus* Ingram & Macfie	-73.1703	-40.5494	0.994	0	13	13
Diptera	*Atrichopogon obnubilus* Ingram & Macfie	-73.1697	-31.0281	0.994	0	13	13
Diptera	*Atrichopogon obfuscatus* Ingram & Macfie	-71.8333	-44.8833	0.992	0	8	8
Diptera	*Atrichopogon obfuscatus* Ingram & Macfie	-72.8647	-49.0372	0.992	0	8	8
Diptera	*Atrichopogon obfuscatus* Ingram & Macfie	-71.4931	-41.5867	0.992	0	8	8
Diptera	*Atrichopogon obfuscatus* Ingram & Macfie	-71.7322	-45.7806	0.992	0	8	8
Diptera	*Atrichopogon obfuscatus* Ingram & Macfie	-72.15	-47.95	0.992	0	8	8
Diptera	*Atrichopogon obfuscatus* Ingram & Macfie	-72.8647	-49.1661	0.992	0	8	8
Diptera	*Atrichopogon obfuscatus* Ingram & Macfie	-72.4958	-41.1041	0.992	0	8	8
Diptera	*Atrichopogon obfuscatus* Ingram & Macfie	-72.5856	-41.1181	0.992	0	8	8
Diptera	*Dasyhelea monticola* Ingram & Macfie	-71.1908	-39.2328	0.892	0	29	20
Diptera	*Dasyhelea monticola* Ingram & Macfie	-71.2833	-38.9	0.892	0	29	20

Diptera	*Dasyhelea monticola* Ingram & Macfie	-71.1331	-38.9165	0.892	0	29	20
Diptera	*Dasyhelea monticola* Ingram & Macfie	-71.1439	-39.8697	0.892	0	29	20
Diptera	*Dasyhelea monticola* Ingram & Macfie	-71.6578	-40.7853	0.892	0	29	20
Diptera	*Dasyhelea monticola* Ingram & Macfie	-71.8156	-41.0261	0.892	0	29	20
Diptera	*Dasyhelea monticola* Ingram & Macfie	-67.4594	-41.1431	0.892	0	29	20
Diptera	*Dasyhelea monticola* Ingram & Macfie	-71.8072	-41.0869	0.892	0	29	20
Diptera	*Dasyhelea monticola* Ingram & Macfie	-71.2972	-41.2633	0.892	0	29	20
Diptera	*Dasyhelea monticola* Ingram & Macfie	-71.8072	-41.0869	0.892	0	29	20
Diptera	*Dasyhelea monticola* Ingram & Macfie	-71.4964	-41.1983	0.892	0	29	20
Diptera	*Dasyhelea monticola* Ingram & Macfie	-71.4975	-41.1936	0.892	0	29	20
Diptera	*Dasyhelea monticola* Ingram & Macfie	-71.4919	-41.1978	0.892	0	29	20
Diptera	*Dasyhelea monticola* Ingram & Macfie	-71.7828	-41.2356	0.892	0	29	20
Diptera	*Dasyhelea monticola* Ingram & Macfie	-71.5156	-41.2703	0.892	0	29	20
Diptera	*Dasyhelea monticola* Ingram & Macfie	-71.4125	-40.6508	0.892	0	29	20
Diptera	*Dasyhelea monticola* Ingram & Macfie	-71.6122	-40.48	0.892	0	29	20
Diptera	*Dasyhelea monticola* Ingram & Macfie	-71.5758	-40.8786	0.892	0	29	20
Diptera	*Dasyhelea monticola* Ingram & Macfie	-71.7897	-40.7128	0.892	0	29	20
Diptera	*Dasyhelea monticola* Ingram & Macfie	-71.7036	-40.6469	0.892	0	29	20
Diptera	*Dasyhelea monticola* Ingram & Macfie	-71.5	-44.9333	0.892	0	29	20
Diptera	*Dasyhelea monticola* Ingram & Macfie	-71.7322	-45.7806	0.892	0	29	20
Diptera	*Dasyhelea monticola* Ingram & Macfie	-71.5347	-42.1481	0.892	0	29	20
Diptera	*Dasyhelea monticola* Ingram & Macfie	-72.4164	-49.1661	0.892	0	29	20
Diptera	*Dasyhelea monticola* Ingram & Macfie	-68.5883	-54.845	0.892	0	29	20
Diptera	*Dasyhelea monticola* Ingram & Macfie	-71.65	-38.6667	0.892	0	29	20
Diptera	*Dasyhelea monticola* Ingram & Macfie	-72.9667	-37.8	0.892	0	29	20
Diptera	*Dasyhelea monticola* Ingram & Macfie	-73.1703	-40.5494	0.892	0	29	20
Diptera	*Dasyhelea monticola* Ingram & Macfie	-71.6667	-41.05	0.892	0	29	20
Diptera	*Dasyhelea andensis* Ingram & Macfie	-64.4	-37.3833	0.967	0	22	12
Diptera	*Dasyhelea andensis* Ingram & Macfie	-71.6667	-40.1167	0.967	0	22	12
Diptera	*Dasyhelea andensis* Ingram & Macfie	-70.39	-37.4167	0.967	0	22	12
Diptera	*Dasyhelea andensis* Ingram & Macfie	-71.7075	-41.2356	0.967	0	22	12
Diptera	*Dasyhelea andensis* Ingram & Macfie	-71.7075	-41.3544	0.967	0	22	12
Diptera	*Dasyhelea andensis* Ingram & Macfie	-71.8156	-41.0261	0.967	0	22	12
Diptera	*Dasyhelea andensis* Ingram & Macfie	-67.5956	-41.1411	0.967	0	22	12
Diptera	*Dasyhelea andensis* Ingram & Macfie	-67.4549	-41.1431	0.967	0	22	12
Diptera	*Dasyhelea andensis* Ingram & Macfie	-71.0094	-41.0606	0.967	0	22	12
Diptera	*Dasyhelea andensis* Ingram & Macfie	-71.3278	-41.1978	0.967	0	22	12
Diptera	*Dasyhelea andensis* Ingram & Macfie	-71.5156	-41.2703	0.967	0	22	12
Diptera	*Dasyhelea andensis* Ingram & Macfie	-71.1344	-40.73	0.967	0	22	12
Diptera	*Dasyhelea andensis* Ingram & Macfie	-71.6122	-40.48	0.967	0	22	12
Diptera	*Dasyhelea andensis* Ingram & Macfie	-71.2925	-40.6897	0.967	0	22	12

Diptera	*Dasyhelea andensis* Ingram & Macfie	-71.7897	-40.7128	0.967	0	22	12
Diptera	*Dasyhelea andensis* Ingram & Macfie	-70.0761	-42.5992	0.967	0	22	12
Diptera	*Dasyhelea andensis* Ingram & Macfie	-72.8647	-49.0372	0.967	0	22	12
Diptera	*Dasyhelea andensis* Ingram & Macfie	-72.9667	-37.8	0.967	0	22	12
Diptera	*Dasyhelea andensis* Ingram & Macfie	-72.9333	-41.4667	0.967	0	22	12
Diptera	*Dasyhelea andensis* Ingram & Macfie	-71.8753	-41.0486	0.967	0	22	12
Diptera	*Dasyhelea andensis* Ingram & Macfie	-71.6667	-41.05	0.967	0	22	12
Diptera	*Dasyhelea andensis* Ingram & Macfie	-71.1331	-38.9165	0.967	0	22	12
Diptera	*Dasyhelea shannoni* Ingram & Macfie	-71.6667	-41.05	0.993	0	12	12
Diptera	*Dasyhelea shannoni* Ingram & Macfie	-71.8753	-41.0486	0.993	0	12	12
Diptera	*Dasyhelea shannoni* Ingram & Macfie	-72.2333	-42.8167	0.993	0	12	12
Diptera	*Dasyhelea shannoni* Ingram & Macfie	-73.8333	-41.8667	0.993	0	12	12
Diptera	*Dasyhelea shannoni* Ingram & Macfie	-72.5378	-41.2067	0.993	0	12	12
Diptera	*Dasyhelea shannoni* Ingram & Macfie	-72.9667	-37.8	0.993	0	12	12
Diptera	*Dasyhelea shannoni* Ingram & Macfie	-72.0667	-45.5667	0.993	0	12	12
Diptera	*Dasyhelea shannoni* Ingram & Macfie	-71.6667	-40.1167	0.993	0	12	12
Diptera	*Dasyhelea shannoni* Ingram & Macfie	-72.8647	-49.0372	0.993	0	12	12
Diptera	*Dasyhelea shannoni* Ingram & Macfie	-71.7367	-41.2411	0.993	0	12	12
Diptera	*Dasyhelea shannoni* Ingram & Macfie	-68.58	-54.8458	0.993	0	12	12
Diptera	*Dasyhelea shannoni* Ingram & Macfie	-71.7897	-40.7128	0.993	0	12	12
Diptera	*Palpomyia subfuscula* Ingram & Macfie	-71.8667	-41.05	0.996	0	12	10
Diptera	*Palpomyia subfuscula* Ingram & Macfie	-71.6667	-40.7833	0.996	0	12	10
Diptera	*Palpomyia subfuscula* Ingram & Macfie	-71.3106	-41.1325	0.996	0	12	10
Diptera	*Palpomyia subfuscula* Ingram & Macfie	-71.7344	-41.3813	0.996	0	12	10
Diptera	*Palpomyia subfuscula* Ingram & Macfie	-71.7075	-41.3544	0.996	0	12	10
Diptera	*Palpomyia subfuscula* Ingram & Macfie	-71.8072	-41.0869	0.996	0	12	10
Diptera	*Palpomyia subfuscula* Ingram & Macfie	-71.7828	-41.2356	0.996	0	12	10
Diptera	*Palpomyia subfuscula* Ingram & Macfie	-71.6997	-36.8	0.996	0	12	10
Diptera	*Palpomyia subfuscula* Ingram & Macfie	-73.1667	-39.4333	0.996	0	12	10
Diptera	*Palpomyia subfuscula* Ingram & Macfie	-72.5667	-39.8667	0.996	0	12	10
Diptera	*Palpomyia subfuscula* Ingram & Macfie	-73.8333	-41.8667	0.996	0	12	10
Diptera	*Palpomyia subfuscula* Ingram & Macfie	-73.8	-42.4667	0.996	0	12	10
Diptera	*Palpomyia mapuche* Spinelli, Grogan & Ronderos	-71.0167	-40.1167	0.993	0	12	12
Diptera	*Palpomyia mapuche* Spinelli, Grogan & Ronderos	-71.6667	-40.1167	0.993	0	12	12
Diptera	*Palpomyia mapuche* Spinelli, Grogan & Ronderos	-71.8	-40.6333	0.993	0	12	12
Diptera	*Palpomyia mapuche* Spinelli, Grogan & Ronderos	-71.6667	-41.7167	0.993	0	12	12
Diptera	*Palpomyia mapuche* Spinelli, Grogan & Ronderos	-71.8156	-41.0261	0.993	0	12	12
Diptera	*Palpomyia mapuche* Spinelli, Grogan & Ronderos	-71.5167	-41.9667	0.993	0	12	12
Diptera	*Palpomyia mapuche* Spinelli, Grogan & Ronderos	-71.8156	-41.2356	0.993	0	12	12
Diptera	*Palpomyia mapuche* Spinelli, Grogan & Ronderos	-73.1975	-39.7072	0.993	0	12	12
Diptera	*Palpomyia mapuche* Spinelli, Grogan & Ronderos	-71.6667	-36.9	0.993	0	12	12

Diptera	*Palpomyia mapuche* Spinelli, Grogan & Ronderos	-73.15	-40.5667	0.993	0	12	12
Diptera	*Palpomyia mapuche* Spinelli, Grogan & Ronderos	-72.2833	-41.3833	0.993	0	12	12
Diptera	*Palpomyia mapuche* Spinelli, Grogan & Ronderos	-73.8667	-42.65	0.993	0	12	12
Diptera	*Stilobezzia (Acanthohelea) rava* Ingram & Macfie	-71.8292	-41.0156	0.985	0	8	7
Diptera	*Stilobezzia (Acanthohelea) rava* Ingram & Macfie	-71.8156	-41.0261	0.985	0	8	7
Diptera	*Stilobezzia (Acanthohelea) rava* Ingram & Macfie	-68.5	-54.8333	0.985	0	8	7
Diptera	*Stilobezzia (Acanthohelea) rava* Ingram & Macfie	-67.8536	-54.6878	0.985	0	8	7
Diptera	*Stilobezzia (Acanthohelea) rava* Ingram & Macfie	-68.5728	-54.8556	0.985	0	8	7
Diptera	*Stilobezzia (Acanthohelea) rava* Ingram & Macfie	-67.1419	-55.8614	0.985	0	8	7
Diptera	*Stilobezzia (Acanthohelea) rava* Ingram & Macfie	-67.25	-55.75	0.985	0	8	7
Diptera	*Stilobezzia (Acanthohelea) rava* Ingram & Macfie	-70.9336	-53.1669	0.985	0	8	7
Hemiptera	*Ectemnostega (Ectemnostega) quadrata* (Signoret)	-69.9435	-32.8057	0.96	0	39	25
Hemiptera	*Ectemnostega (Ectemnostega) quadrata* (Signoret)	-70.1944	-35.8786	0.96	0	39	25
Hemiptera	*Ectemnostega (Ectemnostega) quadrata* (Signoret)	-68.4	-45.9666	0.96	0	39	25
Hemiptera	*Ectemnostega (Ectemnostega) quadrata* (Signoret)	-69.05	-45.55	0.96	0	39	25
Hemiptera	*Ectemnostega (Ectemnostega) quadrata* (Signoret)	-70.7702	-42.9939	0.96	0	39	25
Hemiptera	*Ectemnostega (Ectemnostega) quadrata* (Signoret)	-70.6	-37.966	0.96	0	39	25
Hemiptera	*Ectemnostega (Ectemnostega) quadrata* (Signoret)	-71.3226	-39.1198	0.96	0	39	25
Hemiptera	*Ectemnostega (Ectemnostega) quadrata* (Signoret)	-68.1783	-41.7272	0.96	0	39	25
Hemiptera	*Ectemnostega (Ectemnostega) quadrata* (Signoret)	-68.1922	-41.6844	0.96	0	39	25
Hemiptera	*Ectemnostega (Ectemnostega) quadrata* (Signoret)	-70.4097	-41.7936	0.96	0	39	25
Hemiptera	*Ectemnostega (Ectemnostega) quadrata* (Signoret)	-70.1044	-41.2397	0.96	0	39	25
Hemiptera	*Ectemnostega (Ectemnostega) quadrata* (Signoret)	-68.6236	-41.5017	0.96	0	39	25
Hemiptera	*Ectemnostega (Ectemnostega) quadrata* (Signoret)	-67.4619	-41.0897	0.96	0	39	25
Hemiptera	*Ectemnostega (Ectemnostega) quadrata* (Signoret)	-67.4594	-41.1431	0.96	0	39	25
Hemiptera	*Ectemnostega (Ectemnostega) quadrata* (Signoret)	-71.3031	-41.2187	0.96	0	39	25
Hemiptera	*Ectemnostega (Ectemnostega) quadrata* (Signoret)	-68.2871	-41.6405	0.96	0	39	25
Hemiptera	*Ectemnostega (Ectemnostega) quadrata* (Signoret)	-71.6389	-46.5542	0.96	0	39	25
Hemiptera	*Ectemnostega (Ectemnostega) quadrata* (Signoret)	-71.1833	-48.2333	0.96	0	39	25
Hemiptera	*Ectemnostega (Ectemnostega) quadrata* (Signoret)	-66.4833	-48.4	0.96	0	39	25
Hemiptera	*Ectemnostega (Ectemnostega) quadrata* (Signoret)	-72.4166	-50.2166	0.96	0	39	25
Hemiptera	*Ectemnostega (Ectemnostega) quadrata* (Signoret)	-72.5833	-49.5833	0.96	0	39	25
Hemiptera	*Ectemnostega (Ectemnostega) quadrata* (Signoret)	-71.865	-46.9094	0.96	0	39	25
Hemiptera	*Ectemnostega (Ectemnostega) quadrata* (Signoret)	-71.8272	-47.0881	0.96	0	39	25
Hemiptera	*Ectemnostega (Ectemnostega) quadrata* (Signoret)	-72.4166	-50.15	0.96	0	39	25
Hemiptera	*Ectemnostega (Ectemnostega) quadrata* (Signoret)	-70.25	-48.7666	0.96	0	39	25
Hemiptera	*Ectemnostega (Ectemnostega) quadrata* (Signoret)	-68.3234	-54.7521	0.96	0	39	25
Hemiptera	*Ectemnostega (Ectemnostega) quadrata* (Signoret)	-70.9353	-53.1475	0.96	0	39	25
Hemiptera	*Ectemnostega (Ectemnostega) quadrata* (Signoret)	-70.9164	-53.1544	0.96	0	39	25
Hemiptera	*Ectemnostega (Ectemnostega) quadrata* (Signoret)	-70.20625	-35.1565	0.96	0	39	25
Hemiptera	*Ectemnostega (Ectemnostega) quadrata* (Signoret)	-69.94141667	-35.16452778	0.96	0	39	25

Hemiptera	*Ectemnostega (Ectemnostega) quadrata* (Signoret)	-69.69452778	-35.14634722	0.96	0	39	25
Hemiptera	*Ectemnostega (Ectemnostega) quadrata* (Signoret)	-69.86919444	-35.16008333	0.96	0	39	25
Hemiptera	*Ectemnostega (Ectemnostega) quadrata* (Signoret)	-69.83094444	-36.59094444	0.96	0	39	25
Hemiptera	*Ectemnostega (Ectemnostega) quadrata* (Signoret)	-69.83188889	-36.43311111	0.96	0	39	25
Hemiptera	*Ectemnostega (Ectemnostega) quadrata* (Signoret)	-69.84522222	-36.25488889	0.96	0	39	25
Hemiptera	*Ectemnostega (Ectemnostega) quadrata* (Signoret)	-69.83094444	-36.59094444	0.96	0	39	25
Hemiptera	*Ectemnostega (Ectemnostega) quadrata* (Signoret)	-69.91638889	-34.846	0.96	0	39	25
Hemiptera	*Notonecta (Paranecta) virescens* Blanchard	-67.602325	-39.02521944	0.949	0	21	11
Hemiptera	*Notonecta (Paranecta) virescens* Blanchard	-65.66061944	-39.29627222	0.949	0	21	11
Hemiptera	*Notonecta (Paranecta) virescens* Blanchard	-71.5364	-41.80079444	0.949	0	21	11
Hemiptera	*Notonecta (Paranecta) virescens* Blanchard	-71.55016389	-41.05433889	0.949	0	21	11
Hemiptera	*Notonecta (Paranecta) virescens* Blanchard	-71.31218056	-41.14343333	0.949	0	21	11
Hemiptera	*Notonecta (Paranecta) virescens* Blanchard	-65.70765556	-43.30730278	0.949	0	21	11
Hemiptera	*Notonecta (Paranecta) virescens* Blanchard	-71.07111389	-39.95115278	0.949	0	21	11
Hemiptera	*Notonecta (Paranecta) virescens* Blanchard	-71.74688	-43.170002	0.949	0	21	11
Hemiptera	*Notonecta (Paranecta) virescens* Blanchard	-71.628228	-42.817723	0.949	0	21	11
Hemiptera	*Notonecta (Paranecta) virescens* Blanchard	-70.565938	-33.494764	0.949	0	21	11
Hemiptera	*Notonecta (Paranecta) virescens* Blanchard	-70.554987	-34.248073	0.949	0	21	11
Hemiptera	*Notonecta (Paranecta) virescens* Blanchard	-73.237975	-37.255373	0.949	0	21	11
Hemiptera	*Notonecta (Paranecta) virescens* Blanchard	-72.590129	-38.724555	0.949	0	21	11
Hemiptera	*Notonecta (Paranecta) virescens* Blanchard	-73.734946	-42.280175	0.949	0	21	11
Hemiptera	*Notonecta (Paranecta) virescens* Blanchard	-71.595763	-31.126374	0.949	0	21	11
Hemiptera	*Notonecta (Paranecta) virescens* Blanchard	-72.67747	-37.786635	0.949	0	21	11
Hemiptera	*Notonecta (Paranecta) virescens* Blanchard	-73.122815	-40.568043	0.949	0	21	11
Hemiptera	*Notonecta (Paranecta) virescens* Blanchard	-70.80991	-33.569912	0.949	0	21	11
Hemiptera	*Notonecta (Paranecta) virescens* Blanchard	-72.327855	-39.64906	0.949	0	21	11
Hemiptera	*Notonecta (Paranecta) virescens* Blanchard	-72.049032	-39.501264	0.949	0	21	11
Hemiptera	*Notonecta (Paranecta) virescens* Blanchard	-71.069063	-34.251798	0.949	0	21	11
Hemiptera	*Notonecta (Paranecta) vereertbruggheni* Hungerford	-71.5364	-41.99055556	0.973	0	8	7
Hemiptera	*Notonecta (Paranecta) vereertbruggheni* Hungerford	-67.45961111	-41.14308333	0.973	0	8	7
Hemiptera	*Notonecta (Paranecta) vereertbruggheni* Hungerford	-70.92270278	-39.24097778	0.973	0	8	7
Hemiptera	*Notonecta (Paranecta) vereertbruggheni* Hungerford	-70.85269444	-38.67195833	0.973	0	8	7
Hemiptera	*Notonecta (Paranecta) vereertbruggheni* Hungerford	-71.46384722	-43.07904444	0.973	0	8	7
Hemiptera	*Notonecta (Paranecta) vereertbruggheni* Hungerford	-70.819481	-40.221651	0.973	0	8	7
Hemiptera	*Notonecta (Paranecta) vereertbruggheni* Hungerford	-70.812558	-37.971826	0.973	0	8	7
Hemiptera	*Notonecta (Paranecta) vereertbruggheni* Hungerford	-68.17561	-41.68461	0.973	0	8	7
Hemiptera	*Pseudosaldula bergi* (Haglund)	-69.7936	-52.9597	0.972	0	22	22
Hemiptera	*Pseudosaldula bergi* (Haglund)	-67.6165	-54.9329	0.972	0	22	22
Hemiptera	*Pseudosaldula bergi* (Haglund)	-71.26	-45.56	0.972	0	22	22
Hemiptera	*Pseudosaldula bergi* (Haglund)	-70.43	-44.45	0.972	0	22	22
Hemiptera	*Pseudosaldula bergi* (Haglund)	-68.3	-54.8	0.972	0	22	22

Hemiptera	*Pseudosaldula bergi* (Haglund)	-72.53	-45.4	0.972	0	22	22
Hemiptera	*Pseudosaldula bergi* (Haglund)	-72.58	-44.42	0.972	0	22	22
Hemiptera	*Pseudosaldula bergi* (Haglund)	-72.98	-37.78	0.972	0	22	22
Hemiptera	*Pseudosaldula bergi* (Haglund)	-73.3	-37.9	0.972	0	22	22
Hemiptera	*Pseudosaldula bergi* (Haglund)	-73.5834	-42.529	0.972	0	22	22
Hemiptera	*Pseudosaldula bergi* (Haglund)	-73.8091	-42.6127	0.972	0	22	22
Hemiptera	*Pseudosaldula bergi* (Haglund)	-73.83	-41.87	0.972	0	22	22
Hemiptera	*Pseudosaldula bergi* (Haglund)	-73.98	-42.05	0.972	0	22	22
Hemiptera	*Pseudosaldula bergi* (Haglund)	-73.5983	-41.6178	0.972	0	22	22
Hemiptera	*Pseudosaldula bergi* (Haglund)	-72.7146	-42.9214	0.972	0	22	22
Hemiptera	*Pseudosaldula bergi* (Haglund)	-72.7146	-42.9214	0.972	0	22	22
Hemiptera	*Pseudosaldula bergi* (Haglund)	-72.1833	-40.0183	0.972	0	22	22
Hemiptera	*Pseudosaldula bergi* (Haglund)	-72.1833	-39.8833	0.972	0	22	22
Hemiptera	*Pseudosaldula bergi* (Haglund)	-70.9771	-53.3569	0.972	0	22	22
Hemiptera	*Pseudosaldula bergi* (Haglund)	-70.9166	-53.15	0.972	0	22	22
Hemiptera	*Pseudosaldula bergi* (Haglund)	-70.3667	-53.3	0.972	0	22	22
Hemiptera	*Pseudosaldula bergi* (Haglund)	-72.4259	-51.7587	0.972	0	22	22
Hemiptera	*Pseudosaldula bergi* (Haglund)	-72.74	-51.15	0.972	0	22	22
Hemiptera	*Pseudosaldula bergi* (Haglund)	-71.3833	-34.9833	0.972	0	22	22
Hemiptera	*Sigara (Tropocorixa) egbertae* Hungerford	-71.53529	-39.79166	0.965	0	7	7
Hemiptera	*Sigara (Tropocorixa) egbertae* Hungerford	-70.6	-37.966	0.965	0	7	7
Hemiptera	*Sigara (Tropocorixa) egbertae* Hungerford	-70.880259	-38.658977	0.965	0	7	7
Hemiptera	*Sigara (Tropocorixa) egbertae* Hungerford	-71.3	-41.15	0.965	0	7	7
Hemiptera	*Sigara (Tropocorixa) egbertae* Hungerford	-72.204893	-43.429616	0.965	0	7	7
Hemiptera	*Sigara (Tropocorixa) egbertae* Hungerford	-69.0833	-45.6	0.965	0	7	7
Hemiptera	*Sigara (Tropocorixa) egbertae* Hungerford	-69.05742	-45.56115	0.965	0	7	7
Hemiptera	*Sigara (Tropocorixa) jensenhaarupi* Jaczewski	-70.3167	-42.6333	0.958	0	38	15
Hemiptera	*Sigara (Tropocorixa) jensenhaarupi* Jaczewski	-70.0667	-42.5667	0.958	0	38	15
Hemiptera	*Sigara (Tropocorixa) jensenhaarupi* Jaczewski	-68.6833	-32.9666	0.958	0	38	15
Hemiptera	*Sigara (Tropocorixa) jensenhaarupi* Jaczewski	-68.6833	-32.9666	0.958	0	38	15
Hemiptera	*Sigara (Tropocorixa) jensenhaarupi* Jaczewski	-68.6833	-32.9666	0.958	0	38	15
Hemiptera	*Sigara (Tropocorixa) jensenhaarupi* Jaczewski	-68.6833	-32.9666	0.958	0	38	15
Hemiptera	*Sigara (Tropocorixa) jensenhaarupi* Jaczewski	-69.4378	-32.3686	0.958	0	38	15
Hemiptera	*Sigara (Tropocorixa) jensenhaarupi* Jaczewski	-69.1167	-32.75	0.958	0	38	15
Hemiptera	*Sigara (Tropocorixa) jensenhaarupi* Jaczewski	-69.35	-32.5833	0.958	0	38	15
Hemiptera	*Sigara (Tropocorixa) jensenhaarupi* Jaczewski	-69.1972	-32.9542	0.958	0	38	15
Hemiptera	*Sigara (Tropocorixa) jensenhaarupi* Jaczewski	-68.6122	-32.8919	0.958	0	38	15
Hemiptera	*Sigara (Tropocorixa) jensenhaarupi* Jaczewski	-69.2633	-35.6561	0.958	0	38	15
Hemiptera	*Sigara (Tropocorixa) jensenhaarupi* Jaczewski	-69.2436	-35.6647	0.958	0	38	15
Hemiptera	*Sigara (Tropocorixa) jensenhaarupi* Jaczewski	-69.2172	-35.6653	0.958	0	38	15
Hemiptera	*Sigara (Tropocorixa) jensenhaarupi* Jaczewski	-69.1994	-35.6675	0.958	0	38	15

Hemiptera	*Sigara (Tropocorixa) jensenhaarupi* Jaczewski	-70.1792	-35.8308	0.958	0	38	15
Hemiptera	*Sigara (Tropocorixa) jensenhaarupi* Jaczewski	-69.5317	-35.4758	0.958	0	38	15
Hemiptera	*Sigara (Tropocorixa) jensenhaarupi* Jaczewski	-68.1783	-41.7272	0.958	0	38	15
Hemiptera	*Sigara (Tropocorixa) jensenhaarupi* Jaczewski	-70.61	-39.75	0.958	0	38	15
Hemiptera	*Sigara (Tropocorixa) jensenhaarupi* Jaczewski	-68.1783	-41.7272	0.958	0	38	15
Hemiptera	*Sigara (Tropocorixa) jensenhaarupi* Jaczewski	-70.61	-39.75	0.958	0	38	15
Hemiptera	*Sigara (Tropocorixa) jensenhaarupi* Jaczewski	-67.2633	-40.4967	0.958	0	38	15
Hemiptera	*Sigara (Tropocorixa) jensenhaarupi* Jaczewski	-67.4619	-41.0897	0.958	0	38	15
Hemiptera	*Sigara (Tropocorixa) jensenhaarupi* Jaczewski	-67.4594	-41.1431	0.958	0	38	15
Hemiptera	*Sigara (Tropocorixa) jensenhaarupi* Jaczewski	-67.2633	-48.3469	0.958	0	38	15
Hemiptera	*Sigara (Tropocorixa) jensenhaarupi* Jaczewski	-67.4619	-41.09	0.958	0	38	15
Hemiptera	*Sigara (Tropocorixa) jensenhaarupi* Jaczewski	-67.4597	-41.1431	0.958	0	38	15
Hemiptera	*Sigara (Tropocorixa) jensenhaarupi* Jaczewski	-66.6542	-40.9736	0.958	0	38	15
Hemiptera	*Sigara (Tropocorixa) jensenhaarupi* Jaczewski	-66.1633	-40.6775	0.958	0	38	15
Hemiptera	*Sigara (Tropocorixa) jensenhaarupi* Jaczewski	-65.9875	-41.1981	0.958	0	38	15
Hemiptera	*Sigara (Tropocorixa) jensenhaarupi* Jaczewski	-66.6542	-40.9736	0.958	0	38	15
Hemiptera	*Sigara (Tropocorixa) jensenhaarupi* Jaczewski	-66.0753	-41.4331	0.958	0	38	15
Hemiptera	*Sigara (Tropocorixa) jensenhaarupi* Jaczewski	-66.0758	-41.6633	0.958	0	38	15
Hemiptera	*Sigara (Tropocorixa) jensenhaarupi* Jaczewski	-67.4597	-41.1431	0.958	0	38	15
Hemiptera	*Sigara (Tropocorixa) jensenhaarupi* Jaczewski	-68.195	-41.7272	0.958	0	38	15
Hemiptera	*Sigara (Tropocorixa) jensenhaarupi* Jaczewski	-72.4166	-50.15	0.958	0	38	15
Hemiptera	*Sigara (Tropocorixa) jensenhaarupi* Jaczewski	-65.9101	-38.1489	0.958	0	38	15
Hemiptera	*Sigara (Tropocorixa) jensenhaarupi* Jaczewski	-65.3562	-41.617	0.958	0	38	15
Hemiptera	*Acrophyma cumingii* (Westwood)	-71.5154	-419676	0.993	0	24	14
Hemiptera	*Acrophyma cumingii* (Westwood)	-71.0135	-32.9762	0.993	0	24	14
Hemiptera	*Acrophyma cumingii* (Westwood)	-70.6526	-33.3798	0.993	0	24	14
Hemiptera	*Acrophyma cumingii* (Westwood)	-71.0413	-35.6248	0.993	0	24	14
Hemiptera	*Acrophyma cumingii* (Westwood)	-72.7748	-35.999	0.993	0	24	14
Hemiptera	*Acrophyma cumingii* (Westwood)	-72.7943	-36.1395	0.993	0	24	14
Hemiptera	*Acrophyma cumingii* (Westwood)	-71.5498	-36.5498	0.993	0	24	14
Hemiptera	*Acrophyma cumingii* (Westwood)	-73.0477	-36.834	0.993	0	24	14
Hemiptera	*Acrophyma cumingii* (Westwood)	-72.9443	-37.178	0.993	0	24	14
Hemiptera	*Acrophyma cumingii* (Westwood)	-73.0165	-36.8208	0.993	0	24	14
Hemiptera	*Acrophyma cumingii* (Westwood)	-72.0847	-36.6201	0.993	0	24	14
Hemiptera	*Acrophyma cumingii* (Westwood)	-73.0065	-36.84	0.993	0	24	14
Hemiptera	*Acrophyma cumingii* (Westwood)	-73.2415	-38.1357	0.993	0	24	14
Hemiptera	*Acrophyma cumingii* (Westwood)	-72.3329	-39.6398	0.993	0	24	14
Hemiptera	*Acrophyma cumingii* (Westwood)	-72.0089	-39.5607	0.993	0	24	14
Hemiptera	*Acrophyma cumingii* (Westwood)	-72.4223	-40.7195	0.993	0	24	14
Hemiptera	*Acrophyma cumingii* (Westwood)	-72.5077	-41.4502	0.993	0	24	14
Hemiptera	*Acrophyma cumingii* (Westwood)	-71.6565	-35.4297	0.993	0	24	14

Hemiptera	*Acrophyma cumingii* (Westwood)	-71.597	-35.8466	0.993	0	24	14
Hemiptera	*Acrophyma cumingii* (Westwood)	-70.6879	-32.8375	0.993	0	24	14
Hemiptera	*Acrophyma cumingii* (Westwood)	-71.8848	-38.4429	0.993	0	24	14
Hemiptera	*Acrophyma cumingii* (Westwood)	-72.6283	-38.9537	0.993	0	24	14
Hemiptera	*Acrophyma cumingii* (Westwood)	-72.6025	-40.6856	0.993	0	24	14
Hemiptera	*Acrophyma cumingii* (Westwood)	-73.993	-42.6753	0.993	0	24	14
Hemiptera	*Bergidea atrata* Ashlock	-71.5971	-42.0613	0.996	0.000106	8	8
Hemiptera	*Bergidea atrata* Ashlock	-71.5196	-42.063	0.996	0.000106	8	8
Hemiptera	*Bergidea atrata* Ashlock	-71.157042	-40.157042	0.996	0.000106	8	8
Hemiptera	*Bergidea atrata* Ashlock	-71.3106	-41.132	0.996	0.000106	8	8
Hemiptera	*Bergidea atrata* Ashlock	-73.1672	-37.3422	0.996	0.000106	8	8
Hemiptera	*Bergidea atrata* Ashlock	-71.7343	-36.7951	0.996	0.000106	8	8
Hemiptera	*Bergidea atrata* Ashlock	-71.013	-32.9746	0.996	0.000106	8	8
Hemiptera	*Bergidea atrata* Ashlock	-71.4586	-32.553	0.996	0.000106	8	8
Hemiptera	*Bergidea polychroma* (Spinola)	-71.3984	-41.2063	0.985	0.001463	10	10
Hemiptera	*Bergidea polychroma* (Spinola)	-68.5978	-53.0964	0.985	0.001463	10	10
Hemiptera	*Bergidea polychroma* (Spinola)	-68.3043	-54.798	0.985	0.001463	10	10
Hemiptera	*Bergidea polychroma* (Spinola)	-70.9174	-53.1618	0.985	0.001463	10	10
Hemiptera	*Bergidea polychroma* (Spinola)	-71.4586	-32.553	0.985	0.001463	10	10
Hemiptera	*Bergidea polychroma* (Spinola)	-67.6053	-54.9343	0.985	0.001463	10	10
Hemiptera	*Bergidea polychroma* (Spinola)	-66.8872	-55.0557	0.985	0.001463	10	10
Hemiptera	*Bergidea polychroma* (Spinola)	-74.033	-42.0496	0.985	0.001463	10	10
Hemiptera	*Bergidea polychroma* (Spinola)	-73.9269	-42.6214	0.985	0.001463	10	10
Hemiptera	*Bergidea polychroma* (Spinola)	-71.8673	-41.0449	0.985	0.001463	10	10
Hemiptera	*Cylindrocnema plana* Mayr	-71.6697	-40.1052	0.995	0.000098	6	6
Hemiptera	*Cylindrocnema plana* Mayr	-73.993	-42.6753	0.995	0.000098	6	6
Hemiptera	*Cylindrocnema plana* Mayr	-71.0413	-35.6248	0.995	0.000098	6	6
Hemiptera	*Cylindrocnema plana* Mayr	-72.0847	-36.6201	0.995	0.000098	6	6
Hemiptera	*Cylindrocnema plana* Mayr	-71.673	-38.6908	0.995	0.000098	6	6
Hemiptera	*Cylindrocnema plana* Mayr	-73.9377	-42.6054	0.995	0.000098	6	6
Hemiptera	*Ditomotarsus punctiventris* Spinola	-66.8748	-54.9254	0.983	0.01711	12	12
Hemiptera	*Ditomotarsus punctiventris* Spinola	-70.9465	-53.1544	0.983	0.01711	12	12
Hemiptera	*Ditomotarsus punctiventris* Spinola	-70.9354	-53.1474	0.983	0.01711	12	12
Hemiptera	*Ditomotarsus punctiventris* Spinola	-74.4474	-49.1179	0.983	0.01711	12	12
Hemiptera	*Ditomotarsus punctiventris* Spinola	-67.6167	-54.933	0.983	0.01711	12	12
Hemiptera	*Ditomotarsus punctiventris* Spinola	-73.438	-50.989	0.983	0.01711	12	12
Hemiptera	*Ditomotarsus punctiventris* Spinola	-72.5809	-38.7341	0.983	0.01711	12	12
Hemiptera	*Ditomotarsus punctiventris* Spinola	-68.3328	-54.8076	0.983	0.01711	12	12
Hemiptera	*Ditomotarsus punctiventris* Spinola	-68.5233	-50.0239	0.983	0.01711	12	12
Hemiptera	*Ditomotarsus punctiventris* Spinola	-71.7934	-41.068	0.983	0.01711	12	12
Hemiptera	*Ditomotarsus punctiventris* Spinola	-71.4468	-41.0708	0.983	0.01711	12	12

Hemiptera	*Ditomotarsus punctiventris* Spinola	-71.5407	-40.9413	0.983	0.01711	12	12
Hemiptera	*Idiostolus insularis* (Berg)	-68.044	-54.6157	0.988	0	16	16
Hemiptera	*Idiostolus insularis* (Berg)	-67.7086	-53.7856	0.988	0	16	16
Hemiptera	*Idiostolus insularis* (Berg)	-73.4649	-41.3992	0.988	0	16	16
Hemiptera	*Idiostolus insularis* (Berg)	-72.6131	-40.6606	0.988	0	16	16
Hemiptera	*Idiostolus insularis* (Berg)	-67.7765	-55.2114	0.988	0	16	16
Hemiptera	*Idiostolus insularis* (Berg)	-74.477	-49.1344	0.988	0	16	16
Hemiptera	*Idiostolus insularis* (Berg)	-68.2871	-55.1773	0.988	0	16	16
Hemiptera	*Idiostolus insularis* (Berg)	-71.5882	-54.7248	0.988	0	16	16
Hemiptera	*Idiostolus insularis* (Berg)	-73.05	-41.1163	0.988	0	16	16
Hemiptera	*Idiostolus insularis* (Berg)	-72.496	-51.724	0.988	0	16	16
Hemiptera	*Idiostolus insularis* (Berg)	-67.6053	-54.9343	0.988	0	16	16
Hemiptera	*Idiostolus insularis* (Berg)	-71.9018	-38.438	0.988	0	16	16
Hemiptera	*Idiostolus insularis* (Berg)	-73.6517	-42.34	0.988	0	16	16
Hemiptera	*Idiostolus insularis* (Berg)	-73.7424	-43.3568	0.988	0	16	16
Hemiptera	*Idiostolus insularis* (Berg)	-66.8872	-55.0557	0.988	0	16	16
Hemiptera	*Idiostolus insularis* (Berg)	-74.033	-42.0496	0.988	0	16	16
Hemiptera	*Planois gayi* (Spinola)	-74.4474	-49.1179	0.986	0.000002	14	9
Hemiptera	*Planois gayi* (Spinola)	-73.248	-39.8171	0.986	0.000002	14	9
Hemiptera	*Planois gayi* (Spinola)	-73.993	-42.6753	0.986	0.000002	14	9
Hemiptera	*Planois gayi* (Spinola)	-72.7115	-46.4425	0.986	0.000002	14	9
Hemiptera	*Planois gayi* (Spinola)	-71.673	-38.6908	0.986	0.000002	14	9
Hemiptera	*Planois gayi* (Spinola)	-71.9999	-38.6832	0.986	0.000002	14	9
Hemiptera	*Planois gayi* (Spinola)	-71.3536	-40.1544	0.986	0.000002	14	9
Hemiptera	*Planois gayi* (Spinola)	-71.5081	-40.1522	0.986	0.000002	14	9
Hemiptera	*Planois gayi* (Spinola)	-71.6521	-40.7971	0.986	0.000002	14	9
Hemiptera	*Planois gayi* (Spinola)	-71.4017	-40.6621	0.986	0.000002	14	9
Hemiptera	*Planois gayi* (Spinola)	-71.4468	-41.0708	0.986	0.000002	14	9
Hemiptera	*Planois gayi* (Spinola)	-71.3099	-41.1314	0.986	0.000002	14	9
Hemiptera	*Planois gayi* (Spinola)	-71.5621	-41.0723	0.986	0.000002	14	9
Hemiptera	*Planois gayi* (Spinola)	-68.5233	-50.0239	0.986	0.000002	14	9
Hemiptera	*Sinopla humeralis* Signoret	-71.8848	-38.4429	0.993	0.000004	11	11
Hemiptera	*Sinopla humeralis* Signoret	-71.2325	-32.4552	0.993	0.000004	11	11
Hemiptera	*Sinopla humeralis* Signoret	-71.5062	-31.9093	0.993	0.000004	11	11
Hemiptera	*Sinopla humeralis* Signoret	-71.0413	-35.6248	0.993	0.000004	11	11
Hemiptera	*Sinopla humeralis* Signoret	-71.673	-38.6908	0.993	0.000004	11	11
Hemiptera	*Sinopla humeralis* Signoret	-71.9999	-38.6832	0.993	0.000004	11	11
Hemiptera	*Sinopla humeralis* Signoret	-73.0024	-37.7866	0.993	0.000004	11	11
Hemiptera	*Sinopla humeralis* Signoret	-72.3329	-39.6398	0.993	0.000004	11	11
Hemiptera	*Sinopla humeralis* Signoret	-73.3933	-50.9346	0.993	0.000004	11	11
Hemiptera	*Sinopla humeralis* Signoret	-71.5323	-41.0492	0.993	0.000004	11	11

Hemiptera	*Sinopla humeralis* Signoret	-71.8166	-41.0332	0.993	0.000004	11	11
Hemiptera	*Sinopla perpunctatus* Signoret	-72.7102	-51.1176	0.983	0.003013	19	12
Hemiptera	*Sinopla perpunctatus* Signoret	-67.618	-55.0702	0.983	0.003013	19	12
Hemiptera	*Sinopla perpunctatus* Signoret	-68.2842	-55.1785	0.983	0.003013	19	12
Hemiptera	*Sinopla perpunctatus* Signoret	-70.9194	-53.155	0.983	0.003013	19	12
Hemiptera	*Sinopla perpunctatus* Signoret	-72.0847	-36.6201	0.983	0.003013	19	12
Hemiptera	*Sinopla perpunctatus* Signoret	-71.0413	-35.6248	0.983	0.003013	19	12
Hemiptera	*Sinopla perpunctatus* Signoret	-71.4988	-40.1234	0.983	0.003013	19	12
Hemiptera	*Sinopla perpunctatus* Signoret	-71.3536	-40.1544	0.983	0.003013	19	12
Hemiptera	*Sinopla perpunctatus* Signoret	-66.7448	-54.9754	0.983	0.003013	19	12
Hemiptera	*Sinopla perpunctatus* Signoret	-71.5081	-40.1522	0.983	0.003013	19	12
Hemiptera	*Sinopla perpunctatus* Signoret	-71.5407	-40.9413	0.983	0.003013	19	12
Hemiptera	*Sinopla perpunctatus* Signoret	-71.4017	-40.6621	0.983	0.003013	19	12
Hemiptera	*Sinopla perpunctatus* Signoret	-71.4951	-40.4751	0.983	0.003013	19	12
Hemiptera	*Sinopla perpunctatus* Signoret	-71.6521	-40.7971	0.983	0.003013	19	12
Hemiptera	*Sinopla perpunctatus* Signoret	-71.6461	-40.7629	0.983	0.003013	19	12
Hemiptera	*Sinopla perpunctatus* Signoret	-71.1827	-41.1405	0.983	0.003013	19	12
Hemiptera	*Sinopla perpunctatus* Signoret	-71.5389	-41.2069	0.983	0.003013	19	12
Hemiptera	*Sinopla perpunctatus* Signoret	-71.436	-41.146	0.983	0.003013	19	12
Hemiptera	*Sinopla perpunctatus* Signoret	-68.133	-38.9544	0.983	0.003013	19	12
Hemiptera	*Syzygitis poecilus (Spinola)*	-67.7086	-53.7856	0.978	0.042028	17	17
Hemiptera	*Syzygitis poecilus (Spinola)*	-71.2709	-45.935	0.978	0.042028	17	17
Hemiptera	*Syzygitis poecilus (Spinola)*	-71.3106	-41.132	0.978	0.042028	17	17
Hemiptera	*Syzygitis poecilus (Spinola)*	-71.4438	-39.855	0.978	0.042028	17	17
Hemiptera	*Syzygitis poecilus (Spinola)*	-71.5262	-40.3641	0.978	0.042028	17	17
Hemiptera	*Syzygitis poecilus (Spinola)*	-71.3519	-40.1574	0.978	0.042028	17	17
Hemiptera	*Syzygitis poecilus (Spinola)*	-68.133	-38.9544	0.978	0.042028	17	17
Hemiptera	*Syzygitis poecilus (Spinola)*	-73.7424	-43.3568	0.978	0.042028	17	17
Hemiptera	*Syzygitis poecilus (Spinola)*	-67.6053	-54.9343	0.978	0.042028	17	17
Hemiptera	*Syzygitis poecilus (Spinola)*	-66.8872	-55.0557	0.978	0.042028	17	17
Hemiptera	*Syzygitis poecilus (Spinola)*	-68.2871	-55.1773	0.978	0.042028	17	17
Hemiptera	*Syzygitis poecilus (Spinola)*	-67.7765	-55.2114	0.978	0.042028	17	17
Hemiptera	*Syzygitis poecilus (Spinola)*	-73.045	-36.8199	0.978	0.042028	17	17
Hemiptera	*Syzygitis poecilus (Spinola)*	-71.8673	-41.0449	0.978	0.042028	17	17
Hemiptera	*Syzygitis poecilus (Spinola)*	-72.3363	-39.6433	0.978	0.042028	17	17
Hemiptera	*Syzygitis poecilus (Spinola)*	-70.45	-33.5762	0.978	0.042028	17	17
Hemiptera	*Syzygitis poecilus (Spinola)*	-67.0742	-55.082	0.978	0.042028	17	17
Hemiptera	*Ea australis* Distant	-71.271	-45.9381	0.996	0.000261	9	9
Hemiptera	*Ea australis* Distant	-71.0648	-45.5631	0.996	0.000261	9	9
Hemiptera	*Ea australis* Distant	-72.5001	-50.5994	0.996	0.000261	9	9
Hemiptera	*Ea australis* Distant	-71.5256	-40.3405	0.996	0.000261	9	9

Hemiptera	*Ea australis* Distant	-72.6336	-51.6318	0.996	0.000261	9	9
Hemiptera	*Ea australis* Distant	-72.6456	-51.3326	0.996	0.000261	9	9
Hemiptera	*Ea australis* Distant	-72.6049	-51.5689	0.996	0.000261	9	9
Hemiptera	*Ea australis* Distant	-71.5099	-45.9403	0.996	0.000261	9	9
Hemiptera	*Ea australis* Distant	-71.8191	-41.2121	0.996	0.000261	9	9
Odonata	*Andinagrion peterseni* (Ris)	-71.1965	-42.8505	0.987	0.000005	17	17
Odonata	*Andinagrion peterseni* (Ris)	-70.0475	-41.4429	0.987	0.000005	17	17
Odonata	*Andinagrion peterseni* (Ris)	-68.9846	-41.6631	0.987	0.000005	17	17
Odonata	*Andinagrion peterseni* (Ris)	-70.9763	-40.3895	0.987	0.000005	17	17
Odonata	*Andinagrion peterseni* (Ris)	-71.5125	-40.2268	0.987	0.000005	17	17
Odonata	*Andinagrion peterseni* (Ris)	-71.3689	-39.815	0.987	0.000005	17	17
Odonata	*Andinagrion peterseni* (Ris)	-70.555	-38.5893	0.987	0.000005	17	17
Odonata	*Andinagrion peterseni* (Ris)	-67.9839	-38.5319	0.987	0.000005	17	17
Odonata	*Andinagrion peterseni* (Ris)	-66.0784	-38.9245	0.987	0.000005	17	17
Odonata	*Andinagrion peterseni* (Ris)	-67.237	-40.5906	0.987	0.000005	17	17
Odonata	*Andinagrion peterseni* (Ris)	-67.5434	-41.1652	0.987	0.000005	17	17
Odonata	*Andinagrion peterseni* (Ris)	-66.1741	-41.2992	0.987	0.000005	17	17
Odonata	*Andinagrion peterseni* (Ris)	-65.9252	-41.6152	0.987	0.000005	17	17
Odonata	*Andinagrion peterseni* (Ris)	-66.3273	-41.6823	0.987	0.000005	17	17
Odonata	*Andinagrion peterseni* (Ris)	-66.7391	-41.644	0.987	0.000005	17	17
Odonata	*Andinagrion peterseni* (Ris)	-69.35	-32.58333333	0.987	0.000005	17	17
Odonata	*Andinagrion peterseni* (Ris)	-69.35833333	-32.68683333	0.987	0.000005	17	17
Odonata	*Cyanallagma interruptum* Selys	-70.857	-44.3453	0.980	0.000002	52	26
Odonata	*Cyanallagma interruptum* Selys	-71.409	-44.2311	0.980	0.000002	52	26
Odonata	*Cyanallagma interruptum* Selys	-71.4471	-44.6879	0.980	0.000002	52	26
Odonata	*Cyanallagma interruptum* Selys	-71.8564	-46.3726	0.980	0.000002	52	26
Odonata	*Cyanallagma interruptum* Selys	-72.751	-45.3827	0.980	0.000002	52	26
Odonata	*Cyanallagma interruptum* Selys	-72.6083	-44.7736	0.980	0.000002	52	26
Odonata	*Cyanallagma interruptum* Selys	-72.4655	-44.1264	0.980	0.000002	52	26
Odonata	*Cyanallagma interruptum* Selys	-72.2847	-43.5458	0.980	0.000002	52	26
Odonata	*Cyanallagma interruptum* Selys	-71.8659	-43.3459	0.980	0.000002	52	26
Odonata	*Cyanallagma interruptum* Selys	-71.3424	-43.6505	0.980	0.000002	52	26
Odonata	*Cyanallagma interruptum* Selys	-71.5328	-43.1175	0.980	0.000002	52	26
Odonata	*Cyanallagma interruptum* Selys	-71.8278	-42.7463	0.980	0.000002	52	26
Odonata	*Cyanallagma interruptum* Selys	-71.5994	-42.3751	0.980	0.000002	52	26
Odonata	*Cyanallagma interruptum* Selys	-70.9998	-43.2317	0.980	0.000002	52	26
Odonata	*Cyanallagma interruptum* Selys	-70.6952	-43.0318	0.980	0.000002	52	26
Odonata	*Cyanallagma interruptum* Selys	-71.0949	-43.0033	0.980	0.000002	52	26
Odonata	*Cyanallagma interruptum* Selys	-66.3646	-43.6219	0.980	0.000002	52	26
Odonata	*Cyanallagma interruptum* Selys	-73.6648	-42.1276	0.980	0.000002	52	26
Odonata	*Cyanallagma interruptum* Selys	-72.2847	-50.5985	0.980	0.000002	52	26

Odonata	*Cyanallagma interruptum* Selys	-73.5886	-40.5286	0.980	0.000002	52	26
Odonata	*Cyanallagma interruptum* Selys	-73.1032	-39.6816	0.980	0.000002	52	26
Odonata	*Cyanallagma interruptum* Selys	-72.6273	-38.3491	0.980	0.000002	52	26
Odonata	*Cyanallagma interruptum* Selys	-73.0556	-41.4043	0.980	0.000002	52	26
Odonata	*Cyanallagma interruptum* Selys	-72.475	-41.3757	0.980	0.000002	52	26
Odonata	*Cyanallagma interruptum* Selys	-71.4471	-41.8421	0.980	0.000002	52	26
Odonata	*Cyanallagma interruptum* Selys	-71.6851	-41.5946	0.980	0.000002	52	26
Odonata	*Cyanallagma interruptum* Selys	-71.8278	-41.2996	0.980	0.000002	52	26
Odonata	*Cyanallagma interruptum* Selys	-71.5899	-41.3377	0.980	0.000002	52	26
Odonata	*Cyanallagma interruptum* Selys	-70.3906	-41.7374	0.980	0.000002	52	26
Odonata	*Cyanallagma interruptum* Selys	-70.2003	-41.4328	0.980	0.000002	52	26
Odonata	*Cyanallagma interruptum* Selys	-69.81	-41.252	0.980	0.000002	52	26
Odonata	*Cyanallagma interruptum* Selys	-66.574	-41.5471	0.980	0.000002	52	26
Odonata	*Cyanallagma interruptum* Selys	-66.5835	-41.1283	0.980	0.000002	52	26
Odonata	*Cyanallagma interruptum* Selys	-66.1837	-41.3186	0.980	0.000002	52	26
Odonata	*Cyanallagma interruptum* Selys	-71.5518	-40.7761	0.980	0.000002	52	26
Odonata	*Cyanallagma interruptum* Selys	-71.5804	-40.3288	0.980	0.000002	52	26
Odonata	*Cyanallagma interruptum* Selys	-71.2853	-39.7387	0.980	0.000002	52	26
Odonata	*Cyanallagma interruptum* Selys	-70.4287	-39.3865	0.980	0.000002	52	26
Odonata	*Cyanallagma interruptum* Selys	-71.0474	-39.0534	0.980	0.000002	52	26
Odonata	*Cyanallagma interruptum* Selys	-70.1717	-38.9296	0.980	0.000002	52	26
Odonata	*Cyanallagma interruptum* Selys	-69.9052	-38.7012	0.980	0.000002	52	26
Odonata	*Cyanallagma interruptum* Selys	-70.6666	-38.3776	0.980	0.000002	52	26
Odonata	*Cyanallagma interruptum* Selys	-71.114	-36.7596	0.980	0.000002	52	26
Odonata	*Cyanallagma interruptum* Selys	-70.8475	-37.1974	0.980	0.000002	52	26
Odonata	*Cyanallagma interruptum* Selys	-71.1045	-37.6067	0.980	0.000002	52	26
Odonata	*Cyanallagma interruptum* Selys	-70.8189	-37.9398	0.980	0.000002	52	26
Odonata	*Cyanallagma interruptum* Selys	-72.8843	-37.8161	0.980	0.000002	52	26
Odonata	*Cyanallagma interruptum* Selys	-73.3697	-37.5972	0.980	0.000002	52	26
Odonata	*Cyanallagma interruptum* Selys	-72.989	-36.9595	0.980	0.000002	52	26
Odonata	*Cyanallagma interruptum* Selys	-72.3513	-35.7317	0.980	0.000002	52	26
Odonata	*Cyanallagma interruptum* Selys	-69.4376	-32.3685	0.980	0.000002	52	26
Odonata	*Cyanallagma interruptum* Selys	-69.1972	-32.954	0.980	0.000002	52	26
Odonata	*Gomphomacromia paradoxa* Brauer	-72.3634	-45.9508	0.989	0.000038	30	18
Odonata	*Gomphomacromia paradoxa* Brauer	-72.8535	-45.5953	0.989	0.000038	30	18
Odonata	*Gomphomacromia paradoxa* Brauer	-73.0367	-45.1859	0.989	0.000038	30	18
Odonata	*Gomphomacromia paradoxa* Brauer	-72.4065	-44.3187	0.989	0.000038	30	18
Odonata	*Gomphomacromia paradoxa* Brauer	-71.6416	-44.5881	0.989	0.000038	30	18
Odonata	*Gomphomacromia paradoxa* Brauer	-72.1045	-43.6545	0.989	0.000038	30	18
Odonata	*Gomphomacromia paradoxa* Brauer	-72.1942	-43.479	0.989	0.000038	30	18
Odonata	*Gomphomacromia paradoxa* Brauer	-71.6515	-43.5362	0.989	0.000038	30	18

Odonata	*Gomphomacromia paradoxa* Brauer	-71.6188	-42.9158	0.989	0.000038	30	18
Odonata	*Gomphomacromia paradoxa* Brauer	-71.4678	-42.2751	0.989	0.000038	30	18
Odonata	*Gomphomacromia paradoxa* Brauer	-72.4554	-42.6016	0.989	0.000038	30	18
Odonata	*Gomphomacromia paradoxa* Brauer	-72.2351	-41.414	0.989	0.000038	30	18
Odonata	*Gomphomacromia paradoxa* Brauer	-71.8392	-41.1977	0.989	0.000038	30	18
Odonata	*Gomphomacromia paradoxa* Brauer	-71.6596	-40.8834	0.989	0.000038	30	18
Odonata	*Gomphomacromia paradoxa* Brauer	-70.7577	-39.4591	0.989	0.000038	30	18
Odonata	*Gomphomacromia paradoxa* Brauer	-71.2597	-39.8754	0.989	0.000038	30	18
Odonata	*Gomphomacromia paradoxa* Brauer	-71.3086	-39.7162	0.989	0.000038	30	18
Odonata	*Gomphomacromia paradoxa* Brauer	-72.2391	-39.3285	0.989	0.000038	30	18
Odonata	*Gomphomacromia paradoxa* Brauer	-72.0718	-39.2346	0.989	0.000038	30	18
Odonata	*Gomphomacromia paradoxa* Brauer	-73.1125	-39.1285	0.989	0.000038	30	18
Odonata	*Gomphomacromia paradoxa* Brauer	-70.4149	-39.7774	0.989	0.000038	30	18
Odonata	*Gomphomacromia paradoxa* Brauer	-73.275	-38.804	0.989	0.000038	30	18
Odonata	*Gomphomacromia paradoxa* Brauer	-73.0084	-37.7429	0.989	0.000038	30	18
Odonata	*Gomphomacromia paradoxa* Brauer	-72.6111	-37.7103	0.989	0.000038	30	18
Odonata	*Gomphomacromia paradoxa* Brauer	-72.9811	-36.8995	0.989	0.000038	30	18
Odonata	*Gomphomacromia paradoxa* Brauer	-72.7145	-36.4206	0.989	0.000038	30	18
Odonata	*Gomphomacromia paradoxa* Brauer	-72.5785	-35.9254	0.989	0.000038	30	18
Odonata	*Gomphomacromia paradoxa* Brauer	-71.1474	-34.8371	0.989	0.000038	30	18
Odonata	*Gomphomacromia paradoxa* Brauer	-73.1962	-50.9971	0.989	0.000038	30	18
Odonata	*Gomphomacromia paradoxa* Brauer	-73.5993	-42.6161	0.989	0.000038	30	18
Odonata	*Neopetalia punctata* (Hagen)	-72.5713	-39.9955	0.998	0.000008	7	7
Odonata	*Neopetalia punctata* (Hagen)	-71.5194	-39.9326	0.998	0.000008	7	7
Odonata	*Neopetalia punctata* (Hagen)	-71.5858	-40.3485	0.998	0.000008	7	7
Odonata	*Neopetalia punctata* (Hagen)	-72.1869	-40.7189	0.998	0.000008	7	7
Odonata	*Neopetalia punctata* (Hagen)	-72.1659	-41.0858	0.998	0.000008	7	7
Odonata	*Neopetalia punctata* (Hagen)	-73.1747	-39.7597	0.998	0.000008	7	7
Odonata	*Neopetalia punctata* (Hagen)	-73.3875	-38.3005	0.998	0.000008	7	7
Odonata	*Phenes raptor* Rambur	-71.4401	-39.9003	0.995	0	20	20
Odonata	*Phenes raptor* Rambur	-71.9189	-39.929	0.995	0	20	20
Odonata	*Phenes raptor* Rambur	-72.4647	-40.1205	0.995	0	20	20
Odonata	*Phenes raptor* Rambur	-73.0775	-39.7279	0.995	0	20	20
Odonata	*Phenes raptor* Rambur	-72.6466	-39.1342	0.995	0	20	20
Odonata	*Phenes raptor* Rambur	-73.2595	-38.2724	0.995	0	20	20
Odonata	*Phenes raptor* Rambur	-73.3744	-40.3407	0.995	0	20	20
Odonata	*Phenes raptor* Rambur	-71.4114	-40.3312	0.995	0	20	20
Odonata	*Phenes raptor* Rambur	-72.0625	-40.5993	0.995	0	20	20
Odonata	*Phenes raptor* Rambur	-72.5126	-41.3749	0.995	0	20	20
Odonata	*Phenes raptor* Rambur	-72.1008	-41.7388	0.995	0	20	20
Odonata	*Phenes raptor* Rambur	-71.5359	-42.3516	0.995	0	20	20

Odonata	*Phenes raptor* Rambur	-71.9093	-43.3475	0.995	0	20	20
Odonata	*Phenes raptor* Rambur	-72.4934	-42.8304	0.995	0	20	20
Odonata	*Phenes raptor* Rambur	-73.6521	-41.9973	0.995	0	20	20
Odonata	*Phenes raptor* Rambur	-73.8914	-42.6197	0.995	0	20	20
Odonata	*Phenes raptor* Rambur	-72.2143	-37.4015	0.995	0	20	20
Odonata	*Phenes raptor* Rambur	-72.875	-37.0089	0.995	0	20	20
Odonata	*Phenes raptor* Rambur	-73.1622	-37.5355	0.995	0	20	20
Odonata	*Phenes raptor* Rambur	-70.979	-35.6683	0.995	0	20	20
Odonata	*Rhionaeschna variegata* (Fabricius)	-70.2706	-40.2082	0.965	0.000006	53	20
Odonata	*Rhionaeschna variegata* (Fabricius)	-70.033	-41.4383	0.965	0.000006	53	20
Odonata	*Rhionaeschna variegata* (Fabricius)	-68.9894	-41.6526	0.965	0.000006	53	20
Odonata	*Rhionaeschna variegata* (Fabricius)	-72.3626	-35.7043	0.965	0.000006	53	20
Odonata	*Rhionaeschna variegata* (Fabricius)	-72.1274	-37.6247	0.965	0.000006	53	20
Odonata	*Rhionaeschna variegata* (Fabricius)	-73.1072	-37.3634	0.965	0.000006	53	20
Odonata	*Rhionaeschna variegata* (Fabricius)	-73.3032	-39.9631	0.965	0.000006	53	20
Odonata	*Rhionaeschna variegata* (Fabricius)	-70.9778	-39.0617	0.965	0.000006	53	20
Odonata	*Rhionaeschna variegata* (Fabricius)	-71.3044	-39.741	0.965	0.000006	53	20
Odonata	*Rhionaeschna variegata* (Fabricius)	-71.0431	-40.3812	0.965	0.000006	53	20
Odonata	*Rhionaeschna variegata* (Fabricius)	-71.4873	-40.7078	0.965	0.000006	53	20
Odonata	*Rhionaeschna variegata* (Fabricius)	-72.454	-41.0866	0.965	0.000006	53	20
Odonata	*Rhionaeschna variegata* (Fabricius)	-71.631	-41.2042	0.965	0.000006	53	20
Odonata	*Rhionaeschna variegata* (Fabricius)	-71.4612	-41.7659	0.965	0.000006	53	20
Odonata	*Rhionaeschna variegata* (Fabricius)	-71.7355	-42.4583	0.965	0.000006	53	20
Odonata	*Rhionaeschna variegata* (Fabricius)	-72.3495	-42.4583	0.965	0.000006	53	20
Odonata	*Rhionaeschna variegata* (Fabricius)	-70.8994	-42.8633	0.965	0.000006	53	20
Odonata	*Rhionaeschna variegata* (Fabricius)	-67.2284	-40.6294	0.965	0.000006	53	20
Odonata	*Rhionaeschna variegata* (Fabricius)	-67.5289	-41.1911	0.965	0.000006	53	20
Odonata	*Rhionaeschna variegata* (Fabricius)	-66.7189	-41.6353	0.965	0.000006	53	20
Odonata	*Rhionaeschna variegata* (Fabricius)	-72.2973	-43.5296	0.965	0.000006	53	20
Odonata	*Rhionaeschna variegata* (Fabricius)	-71.3959	-43.4512	0.965	0.000006	53	20
Odonata	*Rhionaeschna variegata* (Fabricius)	-70.6643	-44.5486	0.965	0.000006	53	20
Odonata	*Rhionaeschna variegata* (Fabricius)	-70.1156	-45.1234	0.965	0.000006	53	20
Odonata	*Rhionaeschna variegata* (Fabricius)	-68.2474	-46.9001	0.965	0.000006	53	20
Odonata	*Rhionaeschna variegata* (Fabricius)	-71.3828	-46.7956	0.965	0.000006	53	20
Odonata	*Rhionaeschna variegata* (Fabricius)	-72.5977	-44.4832	0.965	0.000006	53	20
Odonata	*Rhionaeschna variegata* (Fabricius)	-71.6963	-44.6008	0.965	0.000006	53	20
Odonata	*Rhionaeschna variegata* (Fabricius)	-72.7023	-45.3063	0.965	0.000006	53	20
Odonata	*Rhionaeschna variegata* (Fabricius)	-72.2189	-45.4369	0.965	0.000006	53	20
Odonata	*Rhionaeschna variegata* (Fabricius)	-71.7355	-45.5284	0.965	0.000006	53	20
Odonata	*Rhionaeschna variegata* (Fabricius)	-71.9445	-46.4428	0.965	0.000006	53	20
Odonata	*Rhionaeschna variegata* (Fabricius)	-71.0954	-47.9321	0.965	0.000006	53	20

Odonata	*Rhionaeschna variegata* (Fabricius)	-70.403	-48.4024	0.965	0.000006	53	20
Odonata	*Rhionaeschna variegata* (Fabricius)	-70.6512	-48.9119	0.965	0.000006	53	20
Odonata	*Rhionaeschna variegata* (Fabricius)	-71.9315	-48.115	0.965	0.000006	53	20
Odonata	*Rhionaeschna variegata* (Fabricius)	-72.3495	-50.3098	0.965	0.000006	53	20
Odonata	*Rhionaeschna variegata* (Fabricius)	-72.9766	-50.3751	0.965	0.000006	53	20
Odonata	*Rhionaeschna variegata* (Fabricius)	-72.7023	-50.9107	0.965	0.000006	53	20
Odonata	*Rhionaeschna variegata* (Fabricius)	-73.5253	-50.5449	0.965	0.000006	53	20
Odonata	*Rhionaeschna variegata* (Fabricius)	-72.6239	-51.4202	0.965	0.000006	53	20
Odonata	*Rhionaeschna variegata* (Fabricius)	-71.9968	-51.2373	0.965	0.000006	53	20
Odonata	*Rhionaeschna variegata* (Fabricius)	-69.789	-52.0473	0.965	0.000006	53	20
Odonata	*Rhionaeschna variegata* (Fabricius)	-70.9778	-52.6874	0.965	0.000006	53	20
Odonata	*Rhionaeschna variegata* (Fabricius)	-71.0693	-53.3145	0.965	0.000006	53	20
Odonata	*Rhionaeschna variegata* (Fabricius)	-68.6002	-54.02	0.965	0.000006	53	20
Odonata	*Rhionaeschna variegata* (Fabricius)	-67.9992	-54.2028	0.965	0.000006	53	20
Odonata	*Rhionaeschna variegata* (Fabricius)	-68.3128	-54.6078	0.965	0.000006	53	20
Odonata	*Rhionaeschna variegata* (Fabricius)	-67.6596	-54.647	0.965	0.000006	53	20
Odonata	*Rhionaeschna variegata* (Fabricius)	-67.0325	-54.7254	0.965	0.000006	53	20
Odonata	*Rhionaeschna variegata* (Fabricius)	-66.1836	-41.3287	0.965	0.000006	53	20
Odonata	*Rhionaeschna variegata* (Fabricius)	-66.3214	-41.687	0.965	0.000006	53	20
Odonata	*Rhionaeschna variegata* (Fabricius)	-65.919	-41.5988	0.965	0.000006	53	20
Odonata	*Sympetrum villosum* Ris	-72.1812	-41.0092	0.994	0	22	14
Odonata	*Sympetrum villosum* Ris	-72.0487	-40.1809	0.994	0	22	14
Odonata	*Sympetrum villosum* Ris	-73.2304	-39.8606	0.994	0	22	14
Odonata	*Sympetrum villosum* Ris	-71.4523	-40.3134	0.994	0	22	14
Odonata	*Sympetrum villosum* Ris	-71.5186	-40.7552	0.994	0	22	14
Odonata	*Sympetrum villosum* Ris	-71.8499	-40.8877	0.994	0	22	14
Odonata	*Sympetrum villosum* Ris	-71.5628	-41.3074	0.994	0	22	14
Odonata	*Sympetrum villosum* Ris	-71.4302	-41.6719	0.994	0	22	14
Odonata	*Sympetrum villosum* Ris	-72.38	-41.6387	0.994	0	22	14
Odonata	*Sympetrum villosum* Ris	-73.2525	-41.5393	0.994	0	22	14
Odonata	*Sympetrum villosum* Ris	-73.8158	-42.5775	0.994	0	22	14
Odonata	*Sympetrum villosum* Ris	-72.1923	-42.7873	0.994	0	22	14
Odonata	*Sympetrum villosum* Ris	-71.7395	-42.7542	0.994	0	22	14
Odonata	*Sympetrum villosum* Ris	-72.115	-43.1628	0.994	0	22	14
Odonata	*Sympetrum villosum* Ris	-71.7726	-43.6046	0.994	0	22	14
Odonata	*Sympetrum villosum* Ris	-72.3469	-43.9359	0.994	0	22	14
Odonata	*Sympetrum villosum* Ris	-71.364	-44.5433	0.994	0	22	14
Odonata	*Sympetrum villosum* Ris	-72.5236	-44.6538	0.994	0	22	14
Odonata	*Sympetrum villosum* Ris	-72.5236	-45.1507	0.994	0	22	14
Odonata	*Sympetrum villosum* Ris	-72.623	-45.9238	0.994	0	22	14
Odonata	*Sympetrum villosum* Ris	-71.6511	-45.1397	0.994	0	22	14

Odonata	*Sympetrum villosum* Ris	-71.2041	-38.3634	0.994	0	22	14
Orthoptera	*Moluchacris cinerascens* (Philippi)	-70.4456	-25.3506	0.995	0.000127	37	13
Orthoptera	*Moluchacris cinerascens* (Philippi)	-70.4456	-25.3506	0.995	0.000127	37	13
Orthoptera	*Moluchacris cinerascens* (Philippi)	-73.1425	-37.02777778	0.995	0.000127	37	13
Orthoptera	*Moluchacris cinerascens* (Philippi)	-73.11777778	-36.71888889	0.995	0.000127	37	13
Orthoptera	*Moluchacris cinerascens* (Philippi)	-73.1494	-36.9842	0.995	0.000127	37	13
Orthoptera	*Moluchacris cinerascens* (Philippi)	-71.1728	-37.087	0.995	0.000127	37	13
Orthoptera	*Moluchacris cinerascens* (Philippi)	-73.0702	-36.7744	0.995	0.000127	37	13
Orthoptera	*Moluchacris cinerascens* (Philippi)	-72.00527778	-34.38944444	0.995	0.000127	37	13
Orthoptera	*Moluchacris cinerascens* (Philippi)	-72.7474	-37.779	0.995	0.000127	37	13
Orthoptera	*Moluchacris cinerascens* (Philippi)	-71.7107	-35.5952	0.995	0.000127	37	13
Orthoptera	*Moluchacris cinerascens* (Philippi)	-70.72611111	-34.47777778	0.995	0.000127	37	13
Orthoptera	*Moluchacris cinerascens* (Philippi)	-71.11611111	-34.02388889	0.995	0.000127	37	13
Orthoptera	*Moluchacris cinerascens* (Philippi)	-70.9737	-33.9707	0.995	0.000127	37	13
Orthoptera	*Moluchacris cinerascens* (Philippi)	-70.658	-33.2854	0.995	0.000127	37	13
Orthoptera	*Moluchacris cinerascens* (Philippi)	-70.6652	-33.1947	0.995	0.000127	37	13
Orthoptera	*Moluchacris cinerascens* (Philippi)	-70.3774	-33.5014	0.995	0.000127	37	13
Orthoptera	*Moluchacris cinerascens* (Philippi)	-70.45	-34.2167	0.995	0.000127	37	13
Orthoptera	*Moluchacris cinerascens* (Philippi)	-70.85027778	-33.43388889	0.995	0.000127	37	13
Orthoptera	*Moluchacris cinerascens* (Philippi)	-70.6076	-33.4067	0.995	0.000127	37	13
Orthoptera	*Moluchacris cinerascens* (Philippi)	-70.84027778	-33.42861111	0.995	0.000127	37	13
Orthoptera	*Moluchacris cinerascens* (Philippi)	-70.6398	-33.5704	0.995	0.000127	37	13
Orthoptera	*Moluchacris cinerascens* (Philippi)	-70.8806	-33.3045	0.995	0.000127	37	13
Orthoptera	*Moluchacris cinerascens* (Philippi)	-70.6465	-33.4522	0.995	0.000127	37	13
Orthoptera	*Moluchacris cinerascens* (Philippi)	-70.5225	-33.4892	0.995	0.000127	37	13
Orthoptera	*Moluchacris cinerascens* (Philippi)	-70.1333	-32.8333	0.995	0.000127	37	13
Orthoptera	*Moluchacris cinerascens* (Philippi)	-70.83555556	-33.41361111	0.995	0.000127	37	13
Orthoptera	*Moluchacris cinerascens* (Philippi)	-70.5743	-33.4925	0.995	0.000127	37	13
Orthoptera	*Moluchacris cinerascens* (Philippi)	-70.6666	-33.6007	0.995	0.000127	37	13
Orthoptera	*Moluchacris cinerascens* (Philippi)	-71.38	-33.6289	0.995	0.000127	37	13
Orthoptera	*Moluchacris cinerascens* (Philippi)	-70.7695	-33.4627	0.995	0.000127	37	13
Orthoptera	*Moluchacris cinerascens* (Philippi)	-70.5208	-33.4279	0.995	0.000127	37	13
Orthoptera	*Moluchacris cinerascens* (Philippi)	-70.5208	-33.4279	0.995	0.000127	37	13
Orthoptera	*Moluchacris cinerascens* (Philippi)	-70.86055556	-33.60694444	0.995	0.000127	37	13
Orthoptera	*Moluchacris cinerascens* (Philippi)	-70.65944444	-33.66527778	0.995	0.000127	37	13
Orthoptera	*Moluchacris cinerascens* (Philippi)	-71.597	-33.0399	0.995	0.000127	37	13
Orthoptera	*Moluchacris cinerascens* (Philippi)	-71.35	-32.55	0.995	0.000127	37	13
Orthoptera	*Moluchacris cinerascens* (Philippi)	-71.2667	-33.3833	0.995	0.000127	37	13
Orthoptera	*Moluchacris cinerascens* (Philippi)	-71.6487	-33.0407	0.995	0.000127	37	13
Orthoptera	*Moluchacris cinerascens* (Philippi)	-71.6221	-33.0631	0.995	0.000127	37	13
Orthoptera	*Tropidostethus angusticollis* (Blanchard)	-71.361	-40.157	0.996	0	32	21

Orthoptera	*Tropidostethus angusticollis* (Blanchard)	-71.6575	-40.1425	0.996	0	32	21
Orthoptera	*Tropidostethus angusticollis* (Blanchard)	-71.6585	-40.1156	0.996	0	32	21
Orthoptera	*Tropidostethus angusticollis* (Blanchard)	-71.6238	-40.1503	0.996	0	32	21
Orthoptera	*Tropidostethus angusticollis* (Blanchard)	-71.3626	-40.1597	0.996	0	32	21
Orthoptera	*Tropidostethus angusticollis* (Blanchard)	-71.7095	-40.1624	0.996	0	32	21
Orthoptera	*Tropidostethus angusticollis* (Blanchard)	-71.5255	-40.3693	0.996	0	32	21
Orthoptera	*Tropidostethus angusticollis* (Blanchard)	-71.634444	-40.161667	0.996	0	32	21
Orthoptera	*Tropidostethus angusticollis* (Blanchard)	-71.4676	-40.133	0.996	0	32	21
Orthoptera	*Tropidostethus angusticollis* (Blanchard)	-71.7956	-41.0714	0.996	0	32	21
Orthoptera	*Tropidostethus angusticollis* (Blanchard)	-71.631389	-40.155556	0.996	0	32	21
Orthoptera	*Tropidostethus angusticollis* (Blanchard)	-71.7358	-41.2414	0.996	0	32	21
Orthoptera	*Tropidostethus angusticollis* (Blanchard)	-72.68722222	-45.40416667	0.996	0	32	21
Orthoptera	*Tropidostethus angusticollis* (Blanchard)	-72.68222222	-44.73	0.996	0	32	21
Orthoptera	*Tropidostethus angusticollis* (Blanchard)	-72.15694444	-39.48305556	0.996	0	32	21
Orthoptera	*Tropidostethus angusticollis* (Blanchard)	-74.0476	-41.8172	0.996	0	32	21
Orthoptera	*Tropidostethus angusticollis* (Blanchard)	-72.23027778	-39.19861111	0.996	0	32	21
Orthoptera	*Tropidostethus angusticollis* (Blanchard)	-72.19972222	-39.28305556	0.996	0	32	21
Orthoptera	*Tropidostethus angusticollis* (Blanchard)	-73.04861111	-41.12138889	0.996	0	32	21
Orthoptera	*Tropidostethus angusticollis* (Blanchard)	-73.46472222	-41.79277778	0.996	0	32	21
Orthoptera	*Tropidostethus angusticollis* (Blanchard)	-72.39916667	-41.14388889	0.996	0	32	21
Orthoptera	*Tropidostethus angusticollis* (Blanchard)	-72.98555556	-41.32111111	0.996	0	32	21
Orthoptera	*Tropidostethus angusticollis* (Blanchard)	-73.20444444	-39.82388889	0.996	0	32	21
Orthoptera	*Tropidostethus angusticollis* (Blanchard)	-72.7474	-37.779	0.996	0	32	21
Orthoptera	*Tropidostethus angusticollis* (Blanchard)	-72.26138889	-40.65888889	0.996	0	32	21
Orthoptera	*Tropidostethus angusticollis* (Blanchard)	-73.73277778	-40.5875	0.996	0	32	21
Orthoptera	*Tropidostethus angusticollis* (Blanchard)	-73.17611111	-40.58111111	0.996	0	32	21
Orthoptera	*Tropidostethus angusticollis* (Blanchard)	-72.11416667	-40.66694444	0.996	0	32	21
Orthoptera	*Tropidostethus angusticollis* (Blanchard)	-73.70972222	-40.54222222	0.996	0	32	21
Orthoptera	*Tropidostethus angusticollis* (Blanchard)	-72.71027778	-42.94361111	0.996	0	32	21
Orthoptera	*Tropidostethus angusticollis* (Blanchard)	-73.22638889	-39.81194444	0.996	0	32	21
Orthoptera	*Tropidostethus angusticollis* (Blanchard)	-73.25805556	-39.83527778	0.996	0	32	21
Orthoptera	*Aucacris eumera* Hebard	-72.0687	-36.6029	0.992	0	17	17
Orthoptera	*Aucacris eumera* Hebard	-72.1206	-36.5971	0.992	0	17	17
Orthoptera	*Aucacris eumera* Hebard	-71.5462	-36.5544	0.992	0	17	17
Orthoptera	*Aucacris eumera* Hebard	-72.0475	-45.56972222	0.992	0	17	17
Orthoptera	*Aucacris eumera* Hebard	-70.43194444	-34.96277778	0.992	0	17	17
Orthoptera	*Aucacris eumera* Hebard	-70.3019	-29.4	0.992	0	17	17
Orthoptera	*Aucacris eumera* Hebard	-70.7054	-30.0357	0.992	0	17	17
Orthoptera	*Aucacris eumera* Hebard	-71.2697	-35.0517	0.992	0	17	17
Orthoptera	*Aucacris eumera* Hebard	-71.8101	-36.1268	0.992	0	17	17
Orthoptera	*Aucacris eumera* Hebard	-71.5676	-35.8441	0.992	0	17	17

Orthoptera	*Aucacris eumera* Hebard	-71.6343	-35.4152	0.992	0	17	17
Orthoptera	*Aucacris eumera* Hebard	-72.11416667	-40.66694444	0.992	0	17	17
Orthoptera	*Aucacris eumera* Hebard	-70.04388889	-33.77194444	0.992	0	17	17
Orthoptera	*Aucacris eumera* Hebard	-70.25138889	-33.3025	0.992	0	17	17
Orthoptera	*Aucacris eumera* Hebard	-69.95	-33.783	0.992	0	17	17
Orthoptera	*Aucacris eumera* Hebard	-70.50138889	-36.03333333	0.992	0	17	17
Orthoptera	*Aucacris eumera* Hebard	-71.11472222	-35.58527778	0.992	0	17	17
Orthoptera	*Elysiacris ensicornis* Cigliano	-73.2242	-38.0041	0.997	0.000009	13	12
Orthoptera	*Elysiacris ensicornis* Cigliano	-72.7164	-37.8002	0.997	0.000009	13	12
Orthoptera	*Elysiacris ensicornis* Cigliano	-73.3482	-37.4691	0.997	0.000009	13	12
Orthoptera	*Elysiacris ensicornis* Cigliano	-73.0309	-36.8328	0.997	0.000009	13	12
Orthoptera	*Elysiacris ensicornis* Cigliano	-72.2361	-37.7086	0.997	0.000009	13	12
Orthoptera	*Elysiacris ensicornis* Cigliano	-72.7164	-37.8002	0.997	0.000009	13	12
Orthoptera	*Elysiacris ensicornis* Cigliano	-73.1667	-38.7	0.997	0.000009	13	12
Orthoptera	*Elysiacris ensicornis* Cigliano	-72.3857	-39.1399	0.997	0.000009	13	12
Orthoptera	*Elysiacris ensicornis* Cigliano	-72.2405	-39.2823	0.997	0.000009	13	12
Orthoptera	*Elysiacris ensicornis* Cigliano	-73.1535	-37.4234	0.997	0.000009	13	12
Orthoptera	*Elysiacris ensicornis* Cigliano	-73.0428	-40.3012	0.997	0.000009	13	12
Orthoptera	*Elysiacris ensicornis* Cigliano	-72.7416	-35.9593	0.997	0.000009	13	12
Orthoptera	*Elysiacris ensicornis* Cigliano	-72.7167	-35.9833	0.997	0.000009	13	12
Orthoptera	*Elasmoderus wagenknechti* (Liebermann)	-70.48	-25.40722222	0.998	0	14	14
Orthoptera	*Elasmoderus wagenknechti* (Liebermann)	-70.595	-27.85777778	0.998	0	14	14
Orthoptera	*Elasmoderus wagenknechti* (Liebermann)	-71.41027778	-30.58027778	0.998	0	14	14
Orthoptera	*Elasmoderus wagenknechti* (Liebermann)	-70.9924	-31.1784	0.998	0	14	14
Orthoptera	*Elasmoderus wagenknechti* (Liebermann)	-71.05	-31.05	0.998	0	14	14
Orthoptera	*Elasmoderus wagenknechti* (Liebermann)	-70.81777778	-29.82638889	0.998	0	14	14
Orthoptera	*Elasmoderus wagenknechti* (Liebermann)	-71.0183	-31.0016	0.998	0	14	14
Orthoptera	*Elasmoderus wagenknechti* (Liebermann)	-71.5032	-30.252	0.998	0	14	14
Orthoptera	*Elasmoderus wagenknechti* (Liebermann)	-70.75583333	-29.57333333	0.998	0	14	14
Orthoptera	*Elasmoderus wagenknechti* (Liebermann)	-71.25	-30.9166	0.998	0	14	14
Orthoptera	*Elasmoderus wagenknechti* (Liebermann)	-71.2121	-30.5966	0.998	0	14	14
Orthoptera	*Elasmoderus wagenknechti* (Liebermann)	-71.1495	-28.0913	0.998	0	14	14
Orthoptera	*Elasmoderus wagenknechti* (Liebermann)	-70.95777778	-31.08027778	0.998	0	14	14
Orthoptera	*Elasmoderus wagenknechti* (Liebermann)	-71.00166667	-30.74888889	0.998	0	14	14
Orthoptera	*Tetrixocephalus willemsei* Gurney & Liebermann	-70.499194	-42.700439	0.992	0.011663	12	12
Orthoptera	*Tetrixocephalus willemsei* Gurney & Liebermann	-69.5736	-35.4875	0.992	0.011663	12	12
Orthoptera	*Tetrixocephalus willemsei* Gurney & Liebermann	-70.1667	-39.4333	0.992	0.011663	12	12
Orthoptera	*Tetrixocephalus willemsei* Gurney & Liebermann	-71.110625	-39.947917	0.992	0.011663	12	12
Orthoptera	*Tetrixocephalus willemsei* Gurney & Liebermann	-70.3805	-39.0324	0.992	0.011663	12	12
Orthoptera	*Tetrixocephalus willemsei* Gurney & Liebermann	-70.622431	-38.066069	0.992	0.011663	12	12
Orthoptera	*Tetrixocephalus willemsei* Gurney & Liebermann	-70.384	-39.0285	0.992	0.011663	12	12

Orthoptera	*Tetrixocephalus willemsei* Gurney & Liebermann	-71.170144	-40.620717	0.992	0.011663	12	12
Orthoptera	*Tetrixocephalus willemsei* Gurney & Liebermann	-70.6493	-40.559	0.992	0.011663	12	12
Orthoptera	*Tetrixocephalus willemsei* Gurney & Liebermann	-70.2667	-40.0833	0.992	0.011663	12	12
Orthoptera	*Tetrixocephalus willemsei* Gurney & Liebermann	-70.0495	-38.9107	0.992	0.011663	12	12
Orthoptera	*Tetrixocephalus willemsei* Gurney & Liebermann	-70.15611111	-49.5125	0.992	0.011663	12	12
Orthoptera	Tristira magellanica (Bruner)	-68.59888889	-53.10472222	0.996	0.000179	10	10
Orthoptera	*Tristira magellanica* (Bruner)	-72.7159	-50.4018	0.996	0.000179	10	10
Orthoptera	*Tristira magellanica* (Bruner)	-72.79472222	-50.31194444	0.996	0.000179	10	10
Orthoptera	*Tristira magellanica* (Bruner)	-69.2857	-51.6324	0.996	0.000179	10	10
Orthoptera	*Tristira magellanica* (Bruner)	-68.2333	-53.0833	0.996	0.000179	10	10
Orthoptera	*Tristira magellanica* (Bruner)	-70.19888889	-52.62361111	0.996	0.000179	10	10
Orthoptera	*Tristira magellanica* (Bruner)	-72.73277778	-50.87722222	0.996	0.000179	10	10
Orthoptera	*Tristira magellanica* (Bruner)	-71.2725	-52.4214	0.996	0.000179	10	10
Orthoptera	*Tristira magellanica* (Bruner)	-70.9865	-53.481	0.996	0.000179	10	10
Orthoptera	*Tristira magellanica* (Bruner)	-73.12805556	-51.125	0.996	0.000179	10	10
Orthoptera	*Nahuelia rubriventris* Liebermann	-71.2705	-42.8606	0.998	0.001214	9	9
Orthoptera	*Nahuelia rubriventris* Liebermann	-71.2603	-40.2304	0.998	0.001214	9	9
Orthoptera	*Nahuelia rubriventris* Liebermann	-71.5167	-40.5	0.998	0.001214	9	9
Orthoptera	*Nahuelia rubriventris* Liebermann	-71.2318	-40.1829	0.998	0.001214	9	9
Orthoptera	*Nahuelia rubriventris* Liebermann	-71.25	-40.2128	0.998	0.001214	9	9
Orthoptera	*Nahuelia rubriventris* Liebermann	-71.3413	-40.1402	0.998	0.001214	9	9
Orthoptera	*Nahuelia rubriventris* Liebermann	-71.402	-39.6931	0.998	0.001214	9	9
Orthoptera	*Nahuelia rubriventris* Liebermann	-71.5822	-41.1009	0.998	0.001214	9	9
Orthoptera	*Nahuelia rubriventris* Liebermann	-71.5772	-41.0953	0.998	0.001214	9	9
Orthoptera	*Nahuelia rubriventris* Liebermann	-71.5772	-41.0953	0.998	0.001214	9	9
Orthoptera	*Moluchacris nigripes* Cigliano	-73.1538	-36.9838	0.997	0	8	8
Orthoptera	*Moluchacris nigripes* Cigliano	-73.0046	-36.745	0.997	0	8	8
Orthoptera	*Moluchacris nigripes* Cigliano	-72.7474	-37.779	0.997	0	8	8
Orthoptera	*Moluchacris nigripes* Cigliano	-72.4484	-37.9536	0.997	0	8	8
Orthoptera	*Moluchacris nigripes* Cigliano	-72.4085	-35.3424	0.997	0	8	8
Orthoptera	*Moluchacris nigripes* Cigliano	-71.1603	-35.6544	0.997	0	8	8
Orthoptera	*Moluchacris nigripes* Cigliano	-71.1988	-35.6017	0.997	0	8	8
Orthoptera	*Moluchacris nigripes* Cigliano	-71.2198	-33.0751	0.997	0	8	8
Orthoptera	*Conometopus sulcaticollis* (Blanchard)	-72.1402	-36.6153	0.995	0	37	11
Orthoptera	*Conometopus sulcaticollis* (Blanchard)	-71.53305556	-33.58444444	0.995	0	37	11
Orthoptera	*Conometopus sulcaticollis* (Blanchard)	-71.1735	-31.6414	0.995	0	37	11
Orthoptera	*Conometopus sulcaticollis* (Blanchard)	-70.81222222	-34.99972222	0.995	0	37	11
Orthoptera	*Conometopus sulcaticollis* (Blanchard)	-73.03611111	-36.84555556	0.995	0	37	11
Orthoptera	*Conometopus sulcaticollis* (Blanchard)	-71.4926	-31.8704	0.995	0	37	11
Orthoptera	*Conometopus sulcaticollis* (Blanchard)	-71.415531	-30.576783	0.995	0	37	11
Orthoptera	*Conometopus sulcaticollis* (Blanchard)	-71.31472222	-29.28888889	0.995	0	37	11

Orthoptera	*Conometopus sulcaticollis* (Blanchard)	-71.33166667	-29.96944444	0.995	0	37	11
Orthoptera	*Conometopus sulcaticollis* (Blanchard)	-71.4225	-30.2019	0.995	0	37	11
Orthoptera	*Conometopus sulcaticollis* (Blanchard)	-70.61194444	-30.36916667	0.995	0	37	11
Orthoptera	*Conometopus sulcaticollis* (Blanchard)	-71.2667	-30.2833	0.995	0	37	11
Orthoptera	*Conometopus sulcaticollis* (Blanchard)	-70.5056	-30.0369	0.995	0	37	11
Orthoptera	*Conometopus sulcaticollis* (Blanchard)	-71.605	-30.30777778	0.995	0	37	11
Orthoptera	*Conometopus sulcaticollis* (Blanchard)	-70.55194444	-29.97583333	0.995	0	37	11
Orthoptera	*Conometopus sulcaticollis* (Blanchard)	-70.720436	-30.017478	0.995	0	37	11
Orthoptera	*Conometopus sulcaticollis* (Blanchard)	-70.0075	-33.81833333	0.995	0	37	11
Orthoptera	*Conometopus sulcaticollis* (Blanchard)	-71.1728	-37.087	0.995	0	37	11
Orthoptera	*Conometopus sulcaticollis* (Blanchard)	-70.49416667	-33.59805556	0.995	0	37	11
Orthoptera	*Conometopus sulcaticollis* (Blanchard)	-71.7107	-35.5952	0.995	0	37	11
Orthoptera	*Conometopus sulcaticollis* (Blanchard)	-70.515	-34.21166667	0.995	0	37	11
Orthoptera	*Conometopus sulcaticollis* (Blanchard)	-70.55527778	-34.24944444	0.995	0	37	11
Orthoptera	*Conometopus sulcaticollis* (Blanchard)	-70.1499	-33.8153	0.995	0	37	11
Orthoptera	*Conometopus sulcaticollis* (Blanchard)	-70.8856	-33.7552	0.995	0	37	11
Orthoptera	*Conometopus sulcaticollis* (Blanchard)	-70.6398	-33.5704	0.995	0	37	11
Orthoptera	*Conometopus sulcaticollis* (Blanchard)	-70.8927	-33.2871	0.995	0	37	11
Orthoptera	*Conometopus sulcaticollis* (Blanchard)	-70.5259	-33.4857	0.995	0	37	11
Orthoptera	*Conometopus sulcaticollis* (Blanchard)	-70.3546	-33.6453	0.995	0	37	11
Orthoptera	*Conometopus sulcaticollis* (Blanchard)	-71.11472222	-35.58527778	0.995	0	37	11
Orthoptera	*Conometopus sulcaticollis* (Blanchard)	-71.52166667	-33.04	0.995	0	37	11
Orthoptera	*Conometopus sulcaticollis* (Blanchard)	-71.4375	-33.06833333	0.995	0	37	11
Orthoptera	*Conometopus sulcaticollis* (Blanchard)	-71.46916667	-33.16444444	0.995	0	37	11
Orthoptera	*Conometopus sulcaticollis* (Blanchard)	-71.66833333	-33.14027778	0.995	0	37	11
Orthoptera	*Conometopus sulcaticollis* (Blanchard)	-71.4669	-32.8449	0.995	0	37	11
Orthoptera	*Conometopus sulcaticollis* (Blanchard)	-71.53305556	-32.97777778	0.995	0	37	11
Orthoptera	*Conometopus sulcaticollis* (Blanchard)	-71.52472222	-32.9925	0.995	0	37	11
Orthoptera	*Conometopus sulcaticollis* (Blanchard)	-71.5326	-33.0478	0.995	0	37	11
Orthoptera	*Elasmoderus lutescens* (Blanchard)	-71.1495	-28.0913	0.998	0	26	26
Orthoptera	*Elasmoderus lutescens* (Blanchard)	-70.1333	-29.0833	0.998	0	26	26
Orthoptera	*Elasmoderus lutescens* (Blanchard)	-70.3273	-27.3769	0.998	0	26	26
Orthoptera	*Elasmoderus lutescens* (Blanchard)	-71.0172	-29.3037	0.998	0	26	26
Orthoptera	*Elasmoderus lutescens* (Blanchard)	-70.8964	-28.9583	0.998	0	26	26
Orthoptera	*Elasmoderus lutescens* (Blanchard)	-70.7784	-28.5939	0.998	0	26	26
Orthoptera	*Elasmoderus lutescens* (Blanchard)	-70.7691	-28.5783	0.998	0	26	26
Orthoptera	*Elasmoderus lutescens* (Blanchard)	-71.4775	-29.07472222	0.998	0	26	26
Orthoptera	*Elasmoderus lutescens* (Blanchard)	-70.8326	-29.7004	0.998	0	26	26
Orthoptera	*Elasmoderus lutescens* (Blanchard)	-71.0167	-30.4333	0.998	0	26	26
Orthoptera	*Elasmoderus lutescens* (Blanchard)	-71.2493	-29.4345	0.998	0	26	26
Orthoptera	*Elasmoderus lutescens* (Blanchard)	-70.65	-29.3833	0.998	0	26	26

Orthoptera	*Elasmoderus lutescens* (Blanchard)	-71.1735	-31.6414	0.998	0	26	26
Orthoptera	*Elasmoderus lutescens* (Blanchard)	-71.0256	-29.3378	0.998	0	26	26
Orthoptera	*Elasmoderus lutescens* (Blanchard)	-71.25	-30.9166	0.998	0	26	26
Orthoptera	*Elasmoderus lutescens* (Blanchard)	-71.3294	-29.33	0.998	0	26	26
Orthoptera	*Elasmoderus lutescens* (Blanchard)	-70.7417	-29.5527	0.998	0	26	26
Orthoptera	*Elasmoderus lutescens* (Blanchard)	-71.2049	-29.5034	0.998	0	26	26
Orthoptera	*Elasmoderus lutescens* (Blanchard)	-71.184	-30.5997	0.998	0	26	26
Orthoptera	*Elasmoderus lutescens* (Blanchard)	-70.81777778	-29.82638889	0.998	0	26	26
Orthoptera	*Elasmoderus lutescens* (Blanchard)	-71.25166667	-29.90527778	0.998	0	26	26
Orthoptera	*Elasmoderus lutescens* (Blanchard)	-71.48777778	-30.25694444	0.998	0	26	26
Orthoptera	*Elasmoderus lutescens* (Blanchard)	-70.720436	-30.017478	0.998	0	26	26
Orthoptera	*Elasmoderus lutescens* (Blanchard)	-70.65	-28.1333	0.998	0	26	26
Orthoptera	*Elasmoderus lutescens* (Blanchard)	-71.005	-31.18027778	0.998	0	26	26
Orthoptera	*Elasmoderus lutescens* (Blanchard)	-71.1375	-31.07555556	0.998	0	26	26
Orthoptera	*Aucacris bullocki* Rehn	-70.075	-39.08055556	0.991	0.013347	17	13
Orthoptera	*Aucacris bullocki* Rehn	-70.6833	-37.1832	0.991	0.013347	17	13
Orthoptera	*Aucacris bullocki* Rehn	-71.1167	-37.8	0.991	0.013347	17	13
Orthoptera	*Aucacris bullocki* Rehn	-71.108	-38.9527	0.991	0.013347	17	13
Orthoptera	*Aucacris bullocki* Rehn	-70.1994	-38.281	0.991	0.013347	17	13
Orthoptera	*Aucacris bullocki* Rehn	-71.1081	-37.8461	0.991	0.013347	17	13
Orthoptera	*Aucacris bullocki* Rehn	-67.55906	-39.01271	0.991	0.013347	17	13
Orthoptera	*Aucacris bullocki* Rehn	-71.6781	-37.3255	0.991	0.013347	17	13
Orthoptera	*Aucacris bullocki* Rehn	-71.3441	-37.4828	0.991	0.013347	17	13
Orthoptera	*Aucacris bullocki* Rehn	-71.49222222	-36.91472222	0.991	0.013347	17	13
Orthoptera	*Aucacris bullocki* Rehn	-71.40888889	-36.905	0.991	0.013347	17	13
Orthoptera	*Aucacris bullocki* Rehn	-71.4359	-37.8918	0.991	0.013347	17	13
Orthoptera	*Aucacris bullocki* Rehn	-71.62027778	-38.68444444	0.991	0.013347	17	13
Orthoptera	*Aucacris bullocki* Rehn	-71.47444444	-38.56055556	0.991	0.013347	17	13
Orthoptera	*Aucacris bullocki* Rehn	-71.63194444	-39.08055556	0.991	0.013347	17	13
Orthoptera	*Aucacris bullocki* Rehn	-71.89	-38.4324	0.991	0.013347	17	13
Orthoptera	*Aucacris bullocki* Rehn	-73.2476	-39.831	0.991	0.013347	17	13
Orthoptera	*Aucacris bullocki* Rehn	-69.960247	-33.937011	0.991	0.013347	17	13
Orthoptera	*Bufonacris bruchi* Brancsik	-68.9993	-45.5825	0.980	0.000001	17	15
Orthoptera	*Bufonacris bruchi* Brancsik	-70.9139	-43.1487	0.980	0.000001	17	15
Orthoptera	*Bufonacris bruchi* Brancsik	-70.4993	-42.7011	0.980	0.000001	17	15
Orthoptera	*Bufonacris bruchi* Brancsik	-71.4562	-43.8973	0.980	0.000001	17	15
Orthoptera	*Bufonacris bruchi* Brancsik	-69.2746	-43.6842	0.980	0.000001	17	15
Orthoptera	*Bufonacris bruchi* Brancsik	-63.9949	-42.4948	0.980	0.000001	17	15
Orthoptera	*Bufonacris bruchi* Brancsik	-69.0784	-45.5844	0.980	0.000001	17	15
Orthoptera	*Bufonacris bruchi* Brancsik	-65.3113	-43.2298	0.980	0.000001	17	15
Orthoptera	*Bufonacris bruchi* Brancsik	-70.0391	-38.8992	0.980	0.000001	17	15

Orthoptera	*Bufonacris bruchi* Brancsik	-70.2667	-40.0833	0.980	0.000001	17	15
Orthoptera	*Bufonacris bruchi* Brancsik	-70.384	-39.0285	0.980	0.000001	17	15
Orthoptera	*Bufonacris bruchi* Brancsik	-70.055	-38.9224	0.980	0.000001	17	15
Orthoptera	*Bufonacris bruchi* Brancsik	-68.1666	-47.6668	0.980	0.000001	17	15
Orthoptera	*Bufonacris bruchi* Brancsik	-72.0911	-49.614	0.980	0.000001	17	15
Orthoptera	*Bufonacris bruchi* Brancsik	-72.7159	-50.4018	0.980	0.000001	17	15
Orthoptera	*Bufonacris bruchi* Brancsik	-72.8582	-50.1947	0.980	0.000001	17	15
Orthoptera	*Bufonacris bruchi* Brancsik	-72.4773	-46.9108	0.980	0.000001	17	15
Orthoptera	*Neuquenina fictor* (Rehn)	-69.021544	-37.328697	0.991	0.000035	11	11
Orthoptera	*Neuquenina fictor* (Rehn)	-70.1365	-39.4235	0.991	0.000035	11	11
Orthoptera	*Neuquenina fictor* (Rehn)	-69.65	-39.1667	0.991	0.000035	11	11
Orthoptera	*Neuquenina fictor* (Rehn)	-70.1642	-39.4387	0.991	0.000035	11	11
Orthoptera	*Neuquenina fictor* (Rehn)	-70.3361	-39.0536	0.991	0.000035	11	11
Orthoptera	*Neuquenina fictor* (Rehn)	-70.070281	-38.2754	0.991	0.000035	11	11
Orthoptera	*Neuquenina fictor* (Rehn)	-69.291111	-39.518611	0.991	0.000035	11	11
Orthoptera	*Neuquenina fictor* (Rehn)	-70.194711	-38.736003	0.991	0.000035	11	11
Orthoptera	*Neuquenina fictor* (Rehn)	-67.8235	-38.9481	0.991	0.000035	11	11
Orthoptera	*Neuquenina fictor* (Rehn)	-71.3833	-41.15	0.991	0.000035	11	11
Orthoptera	*Neuquenina fictor* (Rehn)	-71.5155	-41.9678	0.991	0.000035	11	11
Orthoptera	*Neuquenina fictor* (Rehn)	-67.5542	-39.0152	0.991	0.000035	11	11
Orthoptera	*Tetrixocephalus sergioi* Ronderos	-70.97666667	-33.00027778	0.999	0.000004	10	10
Orthoptera	*Tetrixocephalus sergioi* Ronderos	-71.00444444	-32.99	0.999	0.000004	10	10
Orthoptera	*Tetrixocephalus sergioi* Ronderos	-70.88916667	-33.15333333	0.999	0.000004	10	10
Orthoptera	*Tetrixocephalus sergioi* Ronderos	-71.17	-34.39666667	0.999	0.000004	10	10
Orthoptera	*Tetrixocephalus sergioi* Ronderos	-71.58527778	-33.52861111	0.999	0.000004	10	10
Orthoptera	*Tetrixocephalus sergioi* Ronderos	-70.0503	-33.8267	0.999	0.000004	10	10
Orthoptera	*Tetrixocephalus sergioi* Ronderos	-70.8806	-33.3045	0.999	0.000004	10	10
Orthoptera	*Tetrixocephalus sergioi* Ronderos	-70.72888889	-33.4025	0.999	0.000004	10	10
Orthoptera	*Tetrixocephalus sergioi* Ronderos	-70.596	-32.8167	0.999	0.000004	10	10
Orthoptera	*Tetrixocephalus sergioi* Ronderos	-71.1704	-33.0086	0.999	0.000004	10	10
Orthoptera	*Bufonacris terrestris* Walker	-68.52527778	-52.32861111	0.998	0.444444	8	8
Orthoptera	*Bufonacris terrestris* Walker	-69.1709	-51.5929	0.998	0.444444	8	8
Orthoptera	*Bufonacris terrestris* Walker	-69.3685	-51.621	0.998	0.444444	8	8
Orthoptera	*Bufonacris terrestris* Walker	-69.1832	-51.649	0.998	0.444444	8	8
Orthoptera	*Bufonacris terrestris* Walker	-70.19888889	-52.62361111	0.998	0.444444	8	8
Orthoptera	*Bufonacris terrestris* Walker	-69.74194444	-52.40444444	0.998	0.444444	8	8
Orthoptera	*Bufonacris terrestris* Walker	-69.4835	-52.151	0.998	0.444444	8	8
Orthoptera	*Bufonacris terrestris* Walker	-70.9865	-53.481	0.998	0.444444	8	8
Orthoptera	*Bufonacris terrestris* Walker	-70.9865	-53.481	0.998	0.444444	8	8
Orthoptera	*Tetrixocephalus chilensis* Ronderos	-71.2157	-34.9713	0.999	0	9	9
Orthoptera	*Tetrixocephalus chilensis* Ronderos	-71.6313	-33.6683	0.999	0	9	9

Orthoptera	*Tetrixocephalus chilensis* Ronderos	-71.6618	-33.4554	0.999	0	9	9
Orthoptera	*Tetrixocephalus chilensis* Ronderos	-71.52361111	-32.92388889	0.999	0	9	9
Orthoptera	*Tetrixocephalus chilensis* Ronderos	-71.48638889	-32.71472222	0.999	0	9	9
Orthoptera	*Tetrixocephalus chilensis* Ronderos	-71.53833333	-32.93277778	0.999	0	9	9
Orthoptera	*Tetrixocephalus chilensis* Ronderos	-71.4669	-32.8449	0.999	0	9	9
Orthoptera	*Tetrixocephalus chilensis* Ronderos	-71.53305556	-32.97777778	0.999	0	9	9
Orthoptera	*Tetrixocephalus chilensis* Ronderos	-71.52861111	-32.82666667	0.999	0	9	9
Orthoptera	*Tebacris nigrisoma* Cigliano	-71.312222	-42.070833	0.988	0	7	7
Orthoptera	*Tebacris nigrisoma* Cigliano	-71.7738	-42.171	0.988	0	7	7
Orthoptera	*Tebacris nigrisoma* Cigliano	-71.1547	-41.048	0.988	0	7	7
Orthoptera	*Tebacris nigrisoma* Cigliano	-71.2895	-41.16	0.988	0	7	7
Orthoptera	*Tebacris nigrisoma* Cigliano	-71.4994	-41.2163	0.988	0	7	7
Orthoptera	*Tebacris nigrisoma* Cigliano	-71.5822	-41.1009	0.988	0	7	7
Orthoptera	*Tebacris nigrisoma* Cigliano	-71.615182	-41.764099	0.988	0	7	7
Orthoptera	*Circacris auris* Ronderos & Cigliano	-70.6748	-43.5091	0.989	1	5	5
Orthoptera	*Circacris auris* Ronderos & Cigliano	-70.9237	-43.0571	0.989	1	5	5
Orthoptera	*Circacris auris* Ronderos & Cigliano	-71.5189	-44.9413	0.989	1	5	5
Orthoptera	*Circacris auris* Ronderos & Cigliano	-71.4562	-43.8973	0.989	1	5	5
Orthoptera	*Circacris auris* Ronderos & Cigliano	-71.1566	-41.2167	0.989	1	5	5
Orthoptera	*Peplacris recutita* Rehn	-71.5995	-33.0688	1	0	5	5
Orthoptera	*Peplacris recutita* Rehn	-71.4996	-33.0428	1	0	5	5
Orthoptera	*Peplacris recutita* Rehn	-71.15	-32.9167	1	0	5	5
Orthoptera	*Peplacris recutita* Rehn	-71.65583333	-33.10805556	1	0	5	5
Orthoptera	*Peplacris recutita* Rehn	-71.3293	-33.0664	1	0	5	5
