# Supplementary material for: The fate of endemic insects of the Andean region under the effect of global warming
Source: PLoS One. 2017 Oct 16;12(10):e0186655. doi: 10.1371/journal.pone.0186655 (PMC5643147; doi:10.1371/journal.pone.0186655)

Plos One

A PROCEDURE TO ELUCIDATE THE FATE OF ENDEMIC INSECTS OF THE ANDEAN REGION UNDER THE EFFECT OF GLOBAL WARMING

Montemayor S.I.; Melo M.C.; Scattolini M.C.; Pocco M.E.; del Río M.G.; Dellapé G.; Scheibler E.E.; Roig S.A.; Cazorla C.G.; Dellapé P.M.

S1 Figs. Boxplots. Comparison of variables with low discrepancy for the Magellanic Subpolar Forest (MSF) and the Valdivian Temperate Forest (VTF) late Pleistocene refugia.

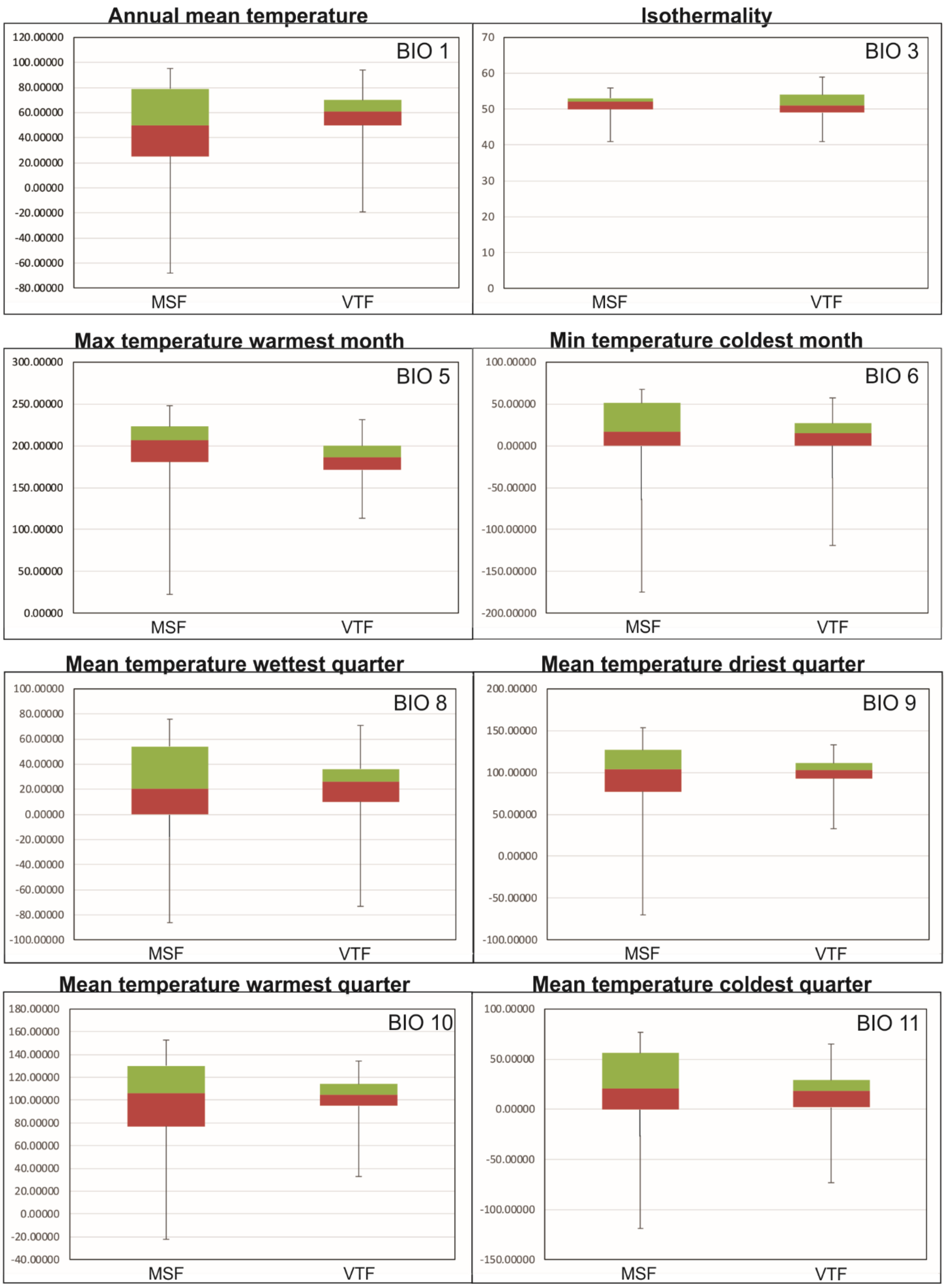

Supplement: S1 Figs — Comparison of variables with low discrepancy for the Magellanic Subpolar Forest (MSF) and the Valdivian Temperate Forest (VTF) Late Pleistocene refugia. (PDF) [file pone.0186655.s002.pdf]
